# Fast-paced and violent media exposure are positively associated with ADHD and impulsivity in college students

**DOI:** 10.3389/fpsyg.2025.1572895

**Published:** 2025-05-12

**Authors:** Nicole L. Hayes, Craig A. Anderson, Edward L. Swing

**Affiliations:** ^1^Worcester Polytechnic Institute, Worcester, MA, United States; ^2^Iowa State University, Ames, IA, United States; ^3^Phoenix Children's Hospital, Phoenix, AZ, United States

**Keywords:** media exposure, media pacing, violent content, ADHD, impulsivity, attention deficits, attention problems

## Abstract

**Introduction:**

Previous research reveals that screen media exposure is positively associated with attention problems and impulsivity.

**Methods:**

Three cross-sectional correlational studies examined the extent to which fast-paced versus violent media exposure are associated with attention-related problems in college students. Multiverse data analyses tested the robustness of results. Fast-paced and violent media effects were examined separately and uniquely using SEM.

**Results:**

A Pilot study (*N* = 233) found weak but significant zero-order correlations of both fast-paced and violent media exposure on self-control. However, the main SEM results were not significant, although in the expected direction, perhaps because of single-item assessments of media pacing and violence. Main Studies 1 (*N =* 438) and 2 (*N =* 456) found that exposure to fast-paced media was positively associated with ADHD-symptoms; this effect was reduced when media violence was added to the model. Both studies also found that greater exposure to violent content was uniquely and positively associated with impulsivity.

**Discussion:**

Overall, findings suggest that fast-paced and violent media yield small but reliable effects that may play an important role in attention-related problems in young adults. Future research should continue to investigate the relationships between media use on attention-related problems, especially longitudinal studies to test direction of causality.

## Introduction

Screen media use has been shown to have both positive and negative associations between social behaviors, as well as cognitive and brain development (see Small et al., 2020 for a review). A growing concern among media effects scholars is the potential harm of high electronic media use on attention and related behavioral problems (e.g., Levine and Waite, 2000; Christakis et al., 2004, 2018), including attention-deficit/hyperactivity disorder (ADHD) symptoms and impulsive behavior. Although attention related problems are often first identified in young children and adolescents, they can persist into adulthood, are comorbid with other psychopathologies (e.g., aggression, anxiety), and are associated with many negative outcomes, including poor school and work performance (Biederman, 2004; Faraone et al., 2006; Gnanavel et al., 2019; Henning et al., 2022; Waschbusch, 2002).

Some scholars suggest that the recent increase in the diagnosis of attention-deficit hyperactivity disorder and severity of symptoms may be attributed to a shift both in media content (more violent, fast-paced, and physiologically arousing) and in the amount of time spent on electronic media (Beyens et al., 2018). While the exact nature and direction of these effects (media or selection effects) are still under investigation, research on media exposure and violent media content has often found small but meaningful associations with attention-related problems (see Beyens et al., 2018; Nikkelen et al., 2014 for reviews). Some research has focused on overall screen time in general, whereas other studies have more specifically focused on violent screen media. One key difficulty in assessing the effects of pacing is that violent video games tend to be very fast-paced. In other words, the pace of video games is confounded with the violent content of video games. More research is needed to examine to what extent pacing contributes to concurrent attention-related problems and to distinguish the violence and pacing risk factors. The present studies including both pacing and violent content measures to allow better tests of the effects of each on attention-related problems.

### Attention-related problems

Attention is a broad construct incorporating various aspects including, but not limited to, visual search, spatial, selective, and sustained attention, which involve attention processes (alerting, orienting, and executive control; Petersen and Posner, 2012). In this article, we focus on attention-related problems related to sustaining attention and executive control, specifically symptoms of Attention Deficit Hyperactivity Disorder (ADHD) and related problems of impulsivity and self-control. ADHD often presents as inattentive, hyper-impulsive, or combined. Individuals with an inattentive presentation have difficulty paying attention to details, are easily distracted, and have difficulty sustaining attention. Individuals with hyper-impulsive presentations have difficulty sitting still for long periods, experience feelings of restlessness, and interrupt or intrude on others (American Psychiatric Association, 2013; Nigg et al., 2010). Individuals with a combined type of ADHD have characteristics of both inattention and hyperactivity.

Impulsivity is a multidimensional construct characterized by poor impulse control, difficulty regulating behavior, and deficits in the delay of gratification. It often is associated with psychopathology and problematic behaviors (Nigg, 2016). A related construct, self-control, is defined as how much control an individual has over their impulses. As noted earlier, such attention problems have been associated with electronic media use in prior studies, including experimental studies (e.g., Hummer et al., 2019; Lillard et al., 2015b; West et al., 2020). How best can such effects be conceptualized?

### Theoretical models of media effects

The Differential Susceptibility to Media Effect Model is useful in understanding how media exposure might contribute to ADHD-type behaviors (DSMM; Valkenburg and Peter, 2013). Accordingly, media effects can be explained by underlying cognitive, emotional, and excitative response states that occur before, during, or after screen media use (see Beyens et al., 2018; Vedechkina and Borgonovi, 2021). Gentile (2011) identified five dimensions along which video games vary, including structure and mechanics of game play, both of which implicate pace. Regarding the role of fast-paced media on ADHD-type behaviors, two hypotheses have been posited: *the scan-and-shift hypothesis* and *the fast-paced arousal-habituation hypothesis*. *The scan-and-shift hypothesis* states that fast-paced media leads to an attentional style of scanning and shifting, Compared to slower-paced media, fast-paced media have frequent cuts, edits, and short scene lengths. High levels of consumption of such media might lead to an attentional style of or preference for scanning and shifting, which involves disengaging, orienting, and re-engaging attention. Over time, the excessive practice of scanning and shifting might lead to difficulty in activities requiring sustained attention. The *fast-paced arousal-habituation hypothesis* states that fast-paced media may lead to increased arousal through repeated attention shifts. In other words, exposure to fast-paced media may affect excitative responses via modulation of arousal levels (Lang et al., 2000). Repeated exposure to fast-paced media may lead to habituation and lower baseline arousal levels, contributing to the development of ADHD-related behaviors. Although these hypotheses propose different mechanisms (e.g., attentional style of scanning and shifting and habituation to arousal) they are not mutually exclusive and may co-occur. The type of media and content may have different impacts on underlying mechanisms involved in attention-related problems including attentional and executive control and reward processing. The scan and shift hypothesis may better explain difficulty sustaining attention as characterized by inattention, whereas the fast-paced arousal hypotheses may be more associated with reward processing and impulsive behaviors.

The DSMM also proposes that media exposure can affect individuals differently based on age, biological sex, overall exposure levels, and exposure to content (Valkenburg and Peter, 2013). These effects may have a differential impact on neural development and cognitive processes depending on developmental age. For example, in young children exposure to fast-paced media may impact neural development in brain regions associated with sustaining attention and control whereas in adolescents and young adults these effects may exacerbate existing issues with sustaining attention and control (Small et al., 2020; see Nigg, 2016 for a review).

### Media exposure and ADHD-related behaviors

Cross-sectional studies have shown that overall media exposure and video game exposure are positively associated with ADHD-related behaviors and impulsivity among children, adolescents, and adults (e.g., Chan and Rabinowitz, 2006; Gentile, 2009). For example, Swing and Anderson (2014) found that both overall screen media use and violent media use were uniquely associated with self-reported attention problems, which were positively associated with aggressive behavior—especially impulsive aggression. Although research on media effects and ADHD-related behaviors has primarily focused on traditional media (TV, movies, and video games), the use of social media applications (e.g., Instagram, Snapchat, and TikTok) has increased in recent years, particularly among adolescents and young adults (Anderson and Jiang, 2018).

Several studies have demonstrated an association between higher levels of social media use and ADHD symptoms (Ra et al., 2018; Boer et al., 2020).

A meta-analysis found a small positive relationship between ADHD behaviors and overall media use (video games and TV) in children and adolescents (Nikkelen et al., 2014). A meta-analysis (Ferguson, 2015) of video games also found small, albeit weaker, effects. This meta-analysis only examined video games, and it (perhaps inappropriately) controlled for a number of factors which necessarily leads to weaker effects.^1^

Several longitudinal studies have found that higher frequency use of television and video games, as well as overall digital media use (e.g., social networking sites, online chatting, texting, streaming television and movies), is longitudinally associated with later ADHD-related problems in children and young adults (Beyens et al., 2020; Christakis et al., 2004, 2018; Gentile et al., 2012, 2016; Swing et al., 2010; Tiraboschi et al., 2022). Some scholars have found a reciprocal relationship between ADHD-related problems and media use with cross-lagged analyses of longitudinal data of children (Beyens et al., 2020). In addition, brief experimental training studies (e.g., 10 h over several weeks) have shown that violent video game training can reduce some attention-related skills (Hummer et al., 2019; West et al., 2020).

### Fast-paced media and ADHD-related behaviors

Most research on media and ADHD-related behaviors has examined either overall screen media exposure or violent media exposure; little research has specifically examined fast-paced media. Several experimental studies investigated whether overstimulation to fast-paced television programs impacted behaviors in children (Anderson et al., 1977; Tower et al., 1979). Results yielded no support for an immediate effect of pacing on subsequent impulsivity and perseverance tasks (Anderson et al., 1977). However, in the latter study, children in the slower-paced TV program, compared to fast-paced TV programs and control group, had relatively greater positive behavior (positive affect and concentration) after two weeks.

Over the past several decades, the pacing of many TV programs has become much faster, and scholars argue that the stimuli used in older studies might underestimate the effects of electronic media use and pacing (Beyens et al., 2018). A few experimental studies with children have investigated the impacts of pacing on ADHD-related behaviors, although these have also yielded mixed results (Cooper et al., 2009; Lillard and Peterson, 2011; Lillard et al., 2015b). Cooper et al. (2009) found that fast-paced TV was associated with fewer attentional problems, whereas Lillard and Peterson (2011) found that children exposed to fast-paced TV, compared to slow-paced TV, had greater attentional problems. Additional experimental research with children has shown that fast-paced and fantastical TV content has short-term effects on executive functions via the depletion of cognitive resources due to stimulating and arousing content (Lillard et al., 2015a; Lillard et al., 2015b).

Although pacing effects have not been directly examined in video game research, several studies have examined neurobehavioral patterns among action video gamers, which often involve fast-paced content. Studies with young adults using event-related potentials (ERP) have found that proactive inhibitory control (e.g., ability to actively maintain goal directed information; associated with anticipation and prevention) is reduced among individuals with higher exposure to action video games (Bailey et al., 2010; Fathi et al., 2022). Differential patterns of activation following violent and nonviolent video game play (both of which were fast paced) have been observed in regions associated with emotion regulation and executive control and increased activation in areas associated with spatial attention and cognitive control regions (Gentile et al., 2016; Wang et al., 2009). Although pacing effects were not directly tested, similarities were also found in regions associated with attentional demands (e.g., selective attention and suppression). These studies suggest that greater exposure to fast-paced media impacts attentional processes, particularly those involved in sustaining attention and maintaining attentional control.

### Present studies

Given the limited research, it is unclear to what extent exposure to fast-paced media is associated with attention related problems (Christakis et al., 2018; Vedechkina and Borgonovi, 2021). We respond to this gap in research by investigating the concurrent associations among exposure to media violence, fast-paced media, and attention-related problems in a series of three cross-sectional studies (labeled Pilot Study, Study 1, and Study 2) using successively larger samples and improved media exposure measures. The Differential Susceptibility to Media Effects Model (DSMM) and prior research on attention-related issues guided our hypotheses and models. Since there is conceptual overlap among attention-related constructs such as ADHD symptoms, impulsivity, and self-control, we also aimed to explore these relationships. We hypothesized that overall exposure to fast-paced media would be positively associated with attention-related problems (ADHD, impulsivity, and self-control). We also examined whether exposure to fast-paced and violent media positively correlate with each other and tested the *unique* associations between media pacing and media violence exposure with attention-related problems. Because biological sex is correlated with both media violence exposure and attention/behavior problems, we included it in some exploratory models (reported in the Supplementary materials (SMs)). We also tested alternative models in which attention related problems predict exposure to fast-paced and violent media. No studies in this manuscript were pre-registered. Data for studies 1 and 2 are available upon request.^2^ Materials and analysis code for all studies are available at: https://osf.io/3pgbt/?view_only=f735dfd3b5904fa68abcde2d56f975e3.

## Pilot study

In a pilot study (*N =* 233) of college students we investigated the extent to which fast-paced and violent media exposure were associated with attention related problems (ADHD symptoms, impulsivity, and self-control). Participants completed a brief media habits questionnaire, measures of ADHD, impulsivity, and self-control (see Supplemental materials) at https://osf.io/3pgbt/?view_only=f735dfd3b5904fa68abcde2d56f975e3). We conducted several SEMs to examine the associations between media content (e.g., violent and fast-paced media) and attention-related problems. First, we tested the hypothesis that exposure to fast-paced media (e.g., video games, TV, and movies) would be positively associated with attention-related problems. Then we added media violence to the model to test whether pacing and violence were uniquely associated with attention-related problems. We also investigated whether biological sex predicted these effects through a series of exploratory SEMs.

This study yielded weak but significant zero-order correlations of both fast-paced and violent media exposure on self-control. However, the main SEM results yielded only non-significant unique paths between the two media exposure variables and, although they were in the expected direction (see Supplementary material). The media variables were highly correlated (*r* = 0.83), suggesting that a larger sample size and better (more distinctive) measures of pacing and violence exposure may be needed to adequately test the unique contributions of each.

## Study 1

Study 1 was a conceptual replication of the Pilot study with a larger sample and improved media exposure measures (6 pacing items per media examples instead of 1; 2 violence items instead of 1).

### Method

#### Participants

In 2020, 497 participants from the same university as in the Pilot study completed an online survey (mean age = 19.40 SD = 1.79, 64% female, 79% Caucasian American, 5.7% Latino American, 3.8% Asian American, 2.9% African American, 1.2% Native American, 5.2% more than ethnicity, and 1% international citizenry).

#### Measures

##### Demographics

Age, biological sex, and ethnicity were collected as part of a background demographic questionnaire (Anderson and Dill, 2000).

##### Media habits

The Media Habits Questionnaire used in the Pilot study was modified by adding additional pacing and violence items. Participants were asked to list their top three frequently played video games, frequently watched television shows, and frequently watched movies, and reported how often they played and/or watched each media example. For each video game, participants were asked to answer two items about violent content (e.g., “How violent is this game?” and “How often do you try to physically injure *players* in this game?”). The first item assessed violent content using the anchors “No violence” (coded as 0) and “Extremely violent” (coded as 8). The second violent content item assessed violent content using anchors “Never” (coded as 0) and “All the time” (coded as 8)^3^ and six items about video game pacing (e.g., This game requires me to make multiple decisions quickly”) using anchors “Never” (coded as 0) and “All the time” (coded as 8). For each television show and movie, participants were asked to answer two items about violent content (e.g., “How often do characters try to physically injure each other”) and four items about media pacing (e.g., “This show/movie requires constant attention to understand what is going on”).

Overall media pacing and media violence exposure scores were computed using valid and recommended practices for assessment of violent and pacing content (see Busching et al., 2015). In accordance with best practices, we computed a separate violence exposure score for each of their three frequently played/watched media by averaging the violent content ratings and multiplying this by the how-often rating^4^. We also computed a separate fast-paced exposure score for each of their three frequently played/watched media by averaging the pacing content ratings and multiplying this by the how-often rating. For each media type (video games, television, movies), violent exposure and pacing scores were averaged to provide overall indices of exposure to violence and fast-paced content for video games, television, and movies. Note that these measures are a Formative type, rather than a Reflective type. This means that there is no underlying assumption that the individual items will be correlated with each other. For example, there is no necessary reason to assume that the pace of a person’s 1st favorite video game should be highly correlated with the pace of their 2nd or 3rd game, or the pace of their other favorite types of media. Therefore, Cronbach’s alpha is not an appropriate measure of reliability (Rose et al., 2023).

##### Attention related problems

###### ADHD

The Adult Attention Deficit-Hyperactivity Disorder Self-Report (ASRS, Brevik et al., 2020) assessed symptoms of ADHD. There are 18 items with three subscales: inattentive (e.g., “How often are you distracted by activity or noise around you?”), hyperactive motor (“How often do you fidget or squirm with your hands or feet when you have to sit down for a long time?”), and hyperactive verbal (e.g., “How often do you interrupt others when they are busy?”). Items were rated on a five-point scale (0 = “Never” to 4 = “Very Often”). Items are averaged for a total score. Higher scores represent greater ADHD symptoms. Reliability for the scores was acceptable: inattentive *α* = 0.83, hyper-motor α = 0.76, hyper-verbal α = 0.83, and total score α = 0.89.

###### Impulsivity

The Urgency, Premeditation, Perseverance, Sensation Seeking, Positive Urgency, Impulsive Behavior Scale (UPPS-P Impulsive Behavior Scale; Whiteside and Lynam, 2001) assessed impulsivity. Items (e.g., “I have trouble controlling my impulses”) are rated on a Likert scale (1 = “Disagree Strongly” to 4 = “Agree Strongly”). Reliability for each of the five subscales was acceptable, ranging from 0.82 to 0.94. See Supplementary materials for details.

###### Self-control

The Brief Self-Control Scale (Tangney et al., 2004) assessed self-control. Participants responded to 13 items (e.g., “I am good at resisting temptation.”). Items were rated on a Likert scale (1 = “Not at All” to 5 = “Very Much”). Several items were reversed scored, then averaged. Reliability was acceptable α = 0.85.

#### Power and precision

To estimate power, we conducted a series of Monte Carlos simulations for SEM with small/medium (0.2) and medium (0.3) effects. Power (0.80) to test hypothesis given a small/medium effect (0.2) in SEM with two latent factors (factor loadings 0.40 (3) and 0.60 (6)), and 4 direct paths requires a sample size of 500. Power (0.80) to test hypothesis given a medium effect (0.3) in SEM with two latent factors (factor loadings 0.40 (3) and 0.60 (6)), and 4 direct paths requires a sample size of 200. Thus Study 1 is well powered to test SEM with small-medium effects for four paths.

#### Data screening and assumptions

We screened the initial sample (*N* = 497) for careless responders and missing data. Twenty-two participants failed attention check questions, and 33 cases had more than 80% missing data and were dropped. Data were then evaluated for potential multivariate and univariate outliers and assumptions of regression. Twenty-seven univariate outliers and four multivariate outliers were identified. Several media variables were slightly positively skewed, although the values were within recommended guidelines; there were no major violations of assumptions.

Analyses were conducted in three ways, consistent with a multiverse approach (Steegen et al., 2016). This multiverse approach resulted in three-overarching analyses: (1) the inclusion of univariate and multivariate outliers, (2) the exclusion of multivariate and univariate outliers, (3) the exclusion of multivariate and transformation of univariate outliers. It was predetermined that if the results did not differ, they would be reported for the model that best fits the data in terms of assumptions. For Study 1, the data excluding multivariate outliers and including univariate outliers transformed (e.g., winsorized) best met all of the regression assumptions and is therefore reported in the results section^5^. Deviations from the results of this found in either of the other two multiverse analyses also are reported in the results section. As in the Pilot study, hypotheses were tested using structural equation modeling (SEM).

#### Planned analyses

First, we examined measurement models with ADHD symptoms, impulsivity, self-control, media violence, and fast-paced media as observed variables. We examined whether attention-related problems best represented a one or two-factor latent structure. After obtaining an acceptable fit for the measurement model, we examined the structural portion of the model by adding paths from media pacing and media violence to endogenous latent variables. Given the complexity of examining models with items as indicators, we used the total scores for media pacing and media violence in subsequent models^6^.

We conducted several SEMs to examine the associations between media content (e.g., violent and fast-paced media) and attention-related problems. First, we tested the hypothesis that exposure to fast-paced media (e.g., video games, TV, and movies) would be positively associated with attention-related problems. Then we added media violence to the model to test whether pacing and violence were uniquely associated with attention-related problems. We also investigated whether biological sex predicted these effects through a series of exploratory SEMs^7^. SEMs were cross validated with 500 bootstrapped samples.^8^

SEMs were conducted in Mplus Version 8.6 using maximum likelihood robust estimation (Muthén and Muthén, 1998–2011). The χ2, comparative fit index (CFI; recommended CFI value ≥ 0.90), Tucker Lewis index (TLI; recommended value ≥ 0.95), standardized root mean square residual (SRMR; recommended SRMR value ≤ 0.08), and root mean square error of approximation (RMSEA; recommended RMSEA value ≤ 0.06) were used as fit indices to test model fit (Hu and Bentler, 1999; Yuan et al., 2016).

Nested models were compared using the Satorra Bentler Scaled χ2 difference test. A statistically significant χ2 difference test rejects the hypothesis of equal model fit. The model that fits the data best is retained (Kline, 2015). In addition to model fit indices, modification indices were also examined to identify areas of poor fit.

### Study 1 results

#### Preliminary analyses

In the preliminary analyses, we examined means, standard deviations, and correlations among variables of interest. In Supplementary Table S2a shows the means and standard deviations, and Supplementary Table S2b shows the zero-order correlations. Of most relevance were significant correlations between: (a) fast-paced media exposure and ADHD (*r =* 0.14) and impulsivity (*r =* 0.15), *ps* < 0.01; (b) violent media exposure and ADHD (*r =* 0.16), self-control (*r =* 0.15), and impulsivity (*r =* 0.19), *ps* < 0.01; and (c) fast-paced and violent media exposure (*r =* 0.56, *p* < 0.001). The latter is considerably smaller than in the Pilot study, suggesting that the additional pacing and violence items led to better (more distinctive) measures of these two concepts.

Males reported higher exposure to media violence than females (*r* = −0.36, *p* < 0.01). The correlations between biological sex and media pacing, total ADHD symptoms, impulsivity, and self-control were non-significant.

#### Attention problems measurement model

A series of confirmatory factor analyses replicated the Pilot study findings of a two-factor model of attention-related problems (see Supplementary materials). The best fit was with ADHD symptoms as one latent factor and impulsivity and self-control as the other latent factor (labelled Impulsivity).

#### Structural equation models

##### Does media pacing predict ADHD-related problems?

**Figure 1:**
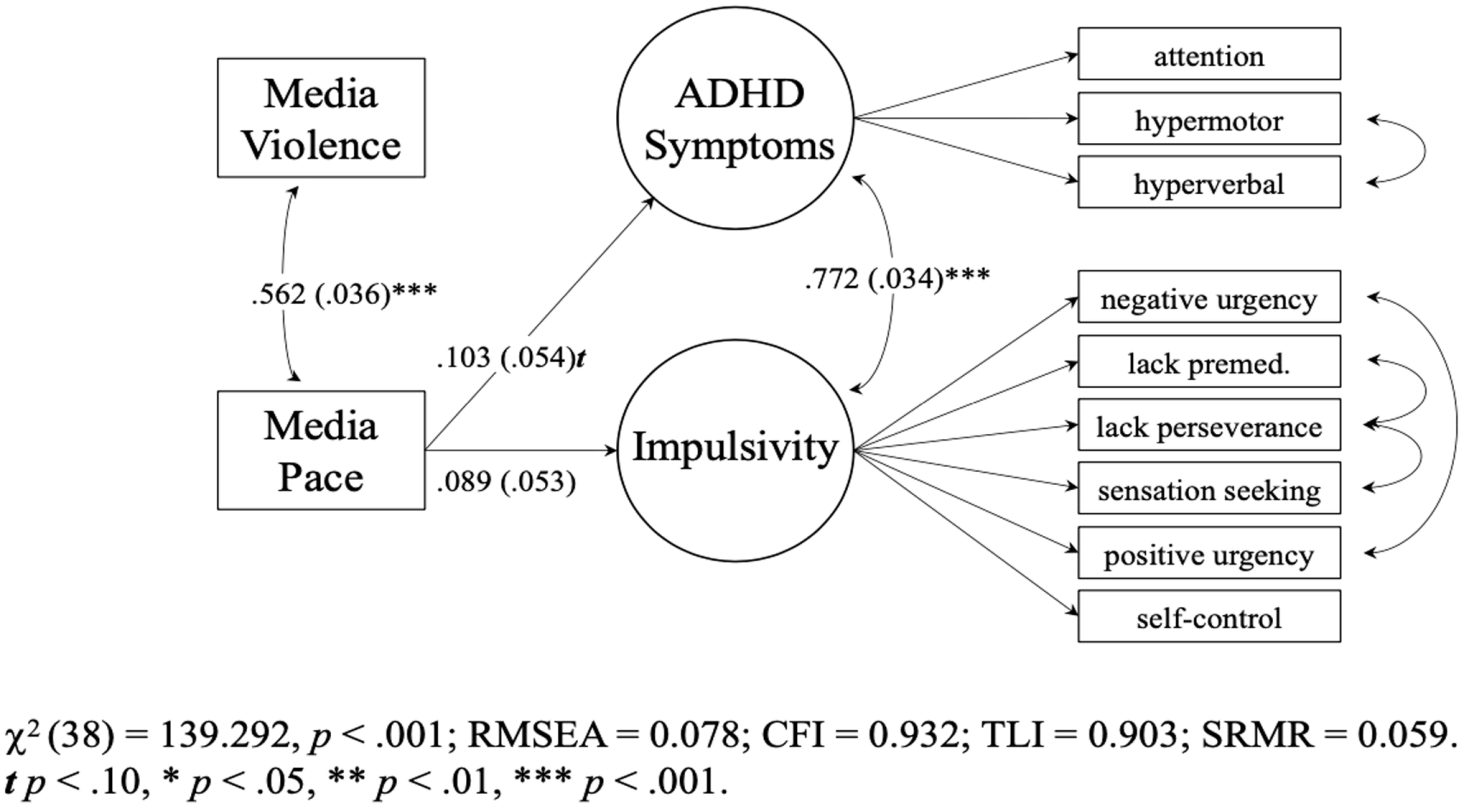
Study 1 (*N* = 438) pacing-only model 6. Parameters are standardized slops. Values in parentheses are SEs. Multiverse approach: media pace->ADHD was significant in outliers included and excluded data sets. Media pace->impulsivity was significant in outliers included date set. See Supplementary materials.

Model 6 (see Supplementary materials) fit the data well, χ2 (38) = 139.292, *p* < 0.001, RMSEA = 0.078 [0.064, 0.092], CFI = 0.932, TLI = 0.903, SRMR = 0.059. Figure 1 shows that media pacing was a marginally significant predictor of ADHD symptoms (B = 0.016 [0.000, 0.033], *p* = 0.056, *β* = 0.103) but not of impulsivity (*B* = 0.011 [−0.002, 0.023], *p* = 0.100, *β* = 0.089).

##### Combined media pacing and media violence model

**Figure 2:**
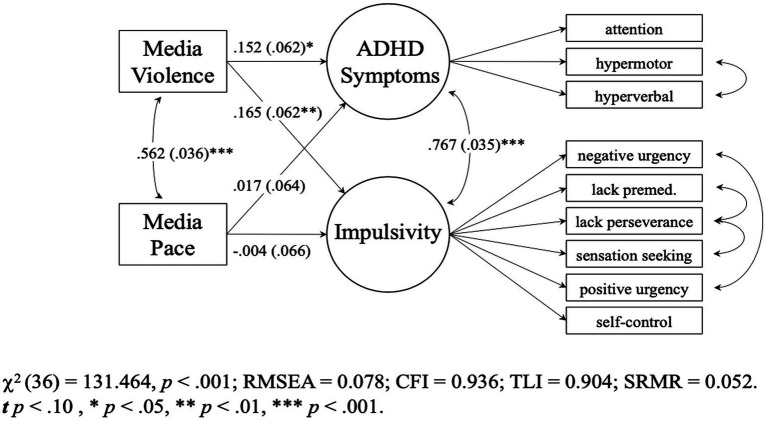
Study 1 (*N* = 438) combined model 7. Parameters are standardized slops. Values in parentheses are SEs. Multiverse approach: media pace->ADHD was significant in outliers included data set. Media violence->ADHD was not significant in outliers included and excluded data sets.

Model 7 fit the data well, χ2 (36) = 131.463 *p* < 0.001, RMSEA = 0.078 [0.064, 0.092], CFI = 0.936 TLI = 0.904, SRMR = 0.052. This combined media pacing and media violence model fit the data better than the media pacing-only model (Model 6), χ2 (2) diff = 7.729, *p* = 0.021 (see Supplementary Table S2e). Figure 2 shows that when media violence was added to the model, media pacing was not a significant unique predictor of either ADHD symptoms (*B* = 0.003 [−0.017, 0.023], *p* = 0.795, *β* = 0.017) or impulsivity (*B* = 0.000 [−0.016, 0.015], *p* = 0.954, *β* = −0.004). Media violence, however, *was* a significant unique predictor of ADHD symptoms (*B* = 0.016 [0.003, 0.028], *p* = 0.016, *β* = 0.152) and impulsivity (*B* = 0.013 [0.003, 0.022], *p* = 0.009, *β* = 0.165). Results were similar when biological sex was added to the model (See Supplementary Figure S2b).

In the alternative direction model, ADHD symptoms and impulsivity were not significant predictors of media pacing and media violence (see Supplementary Figure S2c).

### Study 1 discussion

Study 1 found some evidence that fast-paced media exposure was significantly associated with higher levels of ADHD symptoms. However, when both media variables were in the model, unique pacing effects became non-significant^9^. In contrast, violent media exposure was uniquely positively associated with impulsivity and ADHD symptoms, even after adding sex to the model. Results were mostly consistent across multiverse approaches.

## Study 2

Study 2 attempted to replicate and extend these effects with another large sample from the same university. Most importantly, it improved on our and others’ prior studies by adding assessments of exposure to fast-paced and violent *social media*.

### Method

#### Participants

In 2022, we collected data from five hundred and thirty-one university students (mean age = 19.41 SD = 1.25, 64.5% female, 73.6% Caucasian American, 6.4% Latino American, 6.4% Asian American, 5.8% African American, 2.1% Native American, 0.08%Native Hawaiian/Pacific Islander, 5.2% more than ethnicity, and 3% international citizenry).

#### Measures

##### Demographics

Age, biological sex, and ethnicity were collected as part of a background demographic questionnaire.

##### Media habits

A modified Media Habits Questionnaire assessed exposure to different media types (video games, television, movies, social media) and content (violent, fast-paced vs. slow-paced). The measures were similar to Study 2, with a few exceptions (e.g., one item assessed violent content for each media example listed by participants, five assessed video game pacing, and three assessed TV and movie pacing). The biggest change was including social media items—one violent content item and eight pacing items for each social medium listed by participants (see Supplementary materials for additional details).

Participants were asked to list their top three frequently played video games, watched television shows, watched movies, and used social media applications. They reported how often they played, watched, or used each media example. For each social media application, participants were asked to answer one item about violent content (e.g., “How violent is the content on […]) and eight items about media pacing (e.g., “How often is the content on this social media app/site fast-paced?”). Participants responded using a scale ranging from 1 = “Never” to 7 = “All the Time” for all items. The same procedures were used for video games, TV, and movies to assess pacing and violence exposure. Social media content scores were calculated differently because participants reported time spent on that social media application with an open response, and obtaining an accurate duration was difficult due to multiple response types. For social media, the overall violence and pacing exposure scores were averaged for social media applications and multiplied by the number of hours, on a normal weekday that participants reported spending on social media applications. For each media type (video games, television, movies, social media), violent exposure and pacing scores were transformed to *z*-scores, given that the response format for social media frequency differed from that of video games, TV and movies. These *z*-scores were then averaged to provide overall indices of exposure to violence and fast-paced content for video games, television, movies, and social media.

##### Attention-related problems

The same ADHD (inattentive *α* = 0.82, hyper-motor α = 0.75, hyper-verbal α = 0.80, and total score α = 0.89), self-control (α = 0.82), and impulsivity measures (α = 0.78) were used.

#### Power and precision

Similar to Study 1, to estimate power, we conducted a series of Monte Carlo simulations for SEM. Power (0.80) to test hypothesis given a small/medium effect (0.2) in SEM with two latent factors (factor loadings 0.40 (3) and 0.60 (6), and 4 direct paths requires a sample size of 500). Thus Study 2 is powered to test SEM with small-medium effects for four paths.

#### Data screening and assumptions

Study 2 used the same multiverse approach as in the Pilot and Study 1. We screened the initial sample (*N* = 531) for careless responders and missing data. Thirty-six participants failed attention check questions, and 27 cases had more than 80% missing data; they were deleted. Data were evaluated for potential multivariate and univariate outliers and assumptions of regression using the same procedures. Thirty-two univariate outliers and twelve multivariate outliers were identified. Several media variables were slightly positively skewed, although the values were within recommended guidelines (Kim, 2013); there were no major violations of assumptions. For Study 2, the data set excluding multivariate outliers, and with the univariate outliers transformed (e.g., winsorized), best met the regression assumptions and is therefore reported in this results section.

### Study 2 results

#### Preliminary analyses

We examined means, standard deviations, and correlations among variables of interest (Supplementary Tables S3a,b and Supplementary materials). Of most relevance were significant correlations between: (a) fast-paced exposure and all of the attention problem indicators (*rs* from 0.15 to 0.19, *ps* < 0.01) except for total impulsivity (*r* = 0.03); (b) media violence exposure and all of the attention problem indicators (*rs* from 0.11 to 0.23) except the Hyper-verbal ADHD subscale (*r* = 0.05); and (c) media violence and fast-paced exposure (*r* = 0.58, *p* < 0.001). Males reported higher exposure to media violence than females (*r* = −0.26, *p* < 0.01).

#### Structural equation models

We began by examining and confirming a measurement model in a series of confirmatory factor analyses. A two-factor model of attention-related problems, with ADHD symptoms as one latent factor and impulsivity and self-control as another latent factor, fit the data best.

##### Does media pacing predict ADHD-related problems?

**Figure 3:**
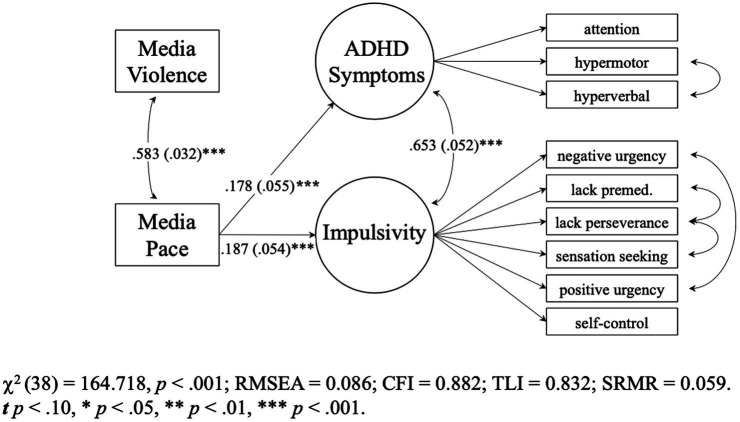
Study 2 (*N* = 456) pacing-only model 6. Parameters are standardized slopes. Values in parentheses are SEs.

Model 6 (see Supplementary materials) adequately fit the data, χ2 (38) = 164.718, *p* < 0.001, RMSEA = 0.086 [0.072, 0.099], CFI = 0.882, TLI = 0.832, SRMR = 0.059. As shown in Figure 3, fast-paced exposure was a significant predictor of ADHD symptoms (*B* = 0.190 [0.078, 0.302], *p* = 0.001, *β* = 0.178) and impulsivity (*B* = 0.124 [0.053, 0.195], *p* = 0.001, *β* = 0.187).

##### Combined media pacing and media violence model

**Figure 4:**
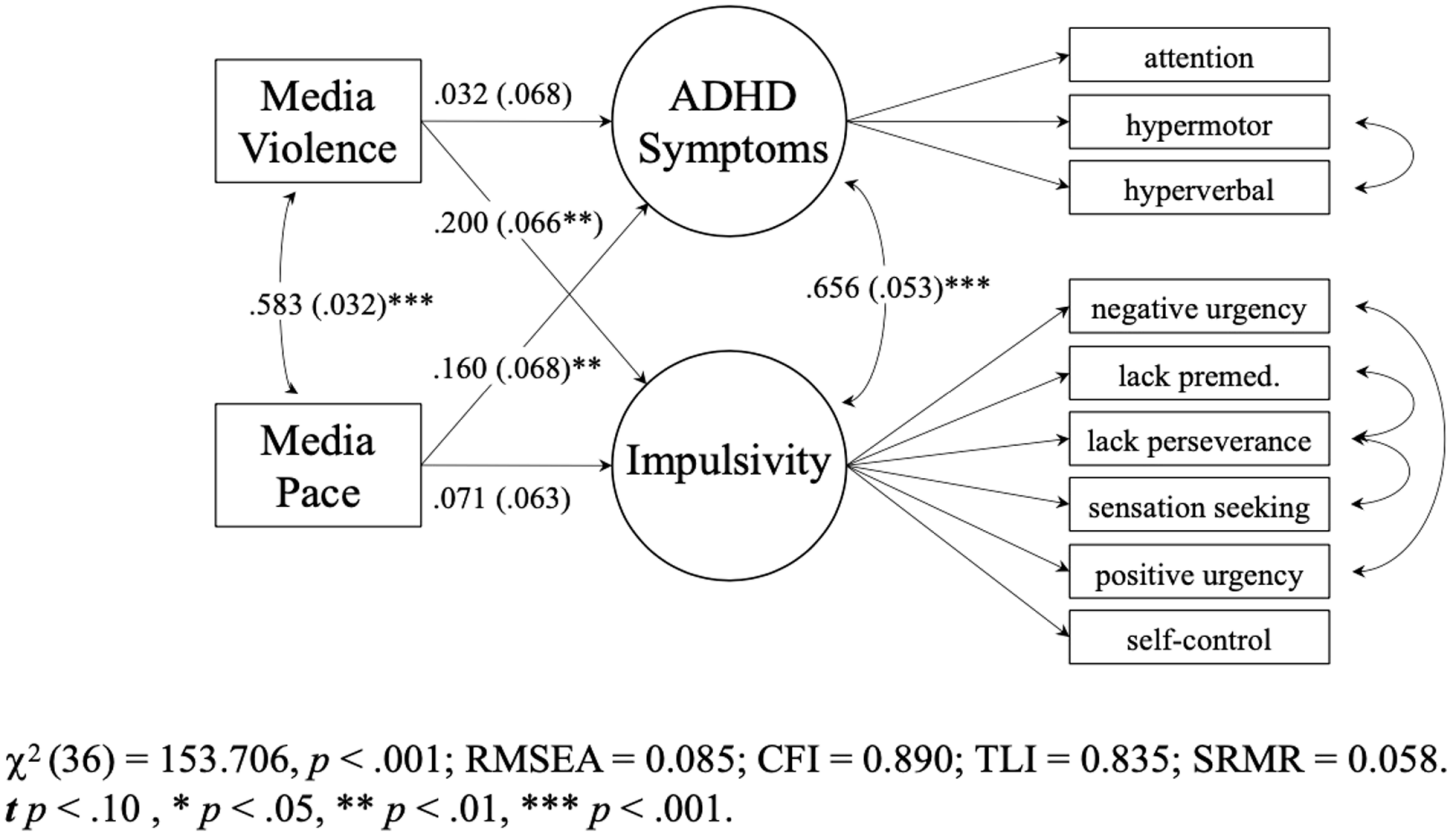
Study 2 (*N* = 456) combined model 7. Parameters are standardized slopes. Values in parentheses are SEs.

Model 7 (see Supplementary materials), which added media violence, also adequately fit the data, χ2 (36) = 153.706, *p* < 0.001, RMSEA = 0.085 [0.071, 0.099], CFI = 0.890 TLI = 0.835, SRMR = 0.058. It fit better than Model 6, χ2 diff (2) = 11.085, *p* = 0.004 (see Supplementary Table S3e). Figure 4 shows that when both media exposure predictors were in the model, fast-paced media exposure uniquely predicted ADHD symptoms (*B* = 0.171 [0.034, 0.308], *p* = 0.015, *β* = 0.160) but not impulsivity (*B* = 0.046 [−0.034, 0.127], *p* = 0.260, *β* = 0.071). Media violence exposure uniquely predicted impulsivity (*B* = 0.127 [0.045, 0.209], *p* = 0.002, *β* = 0.200) but not ADHD symptoms (*B* = 0.033 [−0.107, 0.174], *p* = 0.644, *β* = 0.032). Interestingly, the media pacing effect was reduced to non-significance when biological sex was added to the model, but the media violence effect on impulsivity remained significant (See Supplementary Figure S3b).

In the alternative direction model, ADHD symptoms were neither a significant predictor of media pacing nor of media violence. Impulsivity was not a significant predictor of media pacing, although it significantly predicted media violence (see Supplementary Figure S3c).

### Study 2 discussion

Study 2 found that exposure to fast-paced media was significantly associated with higher levels of ADHD symptoms and impulsivity. When media violence was added to the model, fast-paced media remained a significant unique predictor of ADHD symptoms but not impulsivity. Violent media exposure was uniquely positively associated with impulsivity. It appeared that adding social media pacing and violence variables improved the sensitivity of the media variables. These results were similar across multiverse approaches, highlighting the robustness of these effects.

## Averaged results

We computed weighted average correlations (using Fisher’s *Z* procedure) across the three studies to get a simpler general view of the results. As shown in Table 1, all but two correlations were significant, with the overall *N* = 1,141. Interestingly, media pace was more strongly correlated than was media violence with two of the three ADHD subscales and ADHD Total. Conversely, media violence was more strongly correlated than was media pace with both self-control and impulsivity.

**Table 1:** Weighted average correlations across studies.

	1	2	3	4	5	6	7	8	9
1. Sex	1								
2. Media violence	0.36***	1							
3. Fast-paced media	−0.06*	0.64***	1						
4. Inattention	0.05	0.13***	0.11***	1					
5. Hyper-motor	0.11***	0.11***	0.15***	0.55***	1				
6. Hyper-verbal	0.12***	0.07*	0.14***	0.49***	0.65***	1			
7. ADHD total	0.10***	0.13***	0.16***	0.86***	0.80***	0.76***	1		
8. Self-control	−0.020	0.20***	0.14***	0.59***	0.35***	0.36***	0.55***	1	
9. Impulsivity	−0.06*	0.14***	0.09**	0.48***	0.33***	0.37***	0.49***	0.65***	1

Bolded correlations are significant. ***p < 0.001, **p < 0.01, *p* < 0.05. Sex was coded 0 = male, 1 = female. *N = 1141*.

## General discussion

Across three studies, we investigated the concurrent associations of exposure to fast-paced media and violent media with ADHD-related problems. Patterns among zero-order correlations suggest small to moderate associations between exposure to fast-paced and violent media with ADHD-related problems, with differential impacts on ADHD symptoms versus impulsivity.

Results of structural equation modeling demonstrated that when analyzed separately, individuals with higher exposure to fast-paced media report higher levels of self-reported ADHD-related problems, particularly ADHD symptoms. However, when effects of fast-paced media and media violence are jointly examined, the unique effects of media pacing were reduced. Across Study 1 and Study 2, only media violence yielded significant *unique* effects on self-reported ADHD-related problems in the combined models. However, in Study 2, which added social media measures, individuals with higher exposure to fast-paced media reported significantly higher levels of ADHD- symptoms even when media violence was included in the model. Results were similar across models and multiverse approaches.

These are the first studies to directly compare media pacing and media violence exposure effects on concurrent attention-related problems. The findings were a bit surprising in that we expected the media pacing-to-attention problems association to be at least as strong as the media violence-to-attention problems link, but it was not. Nonetheless, the results support the theoretical prediction from Gentile (2011) that pacing should affect attention processes, skills, and habits. One reasonable explanation for the observed positive association between screen media and attention problems is the scan-and-shift hypothesis which states that high exposure to fast-paced media requires constant switching of one’s attention, perhaps leading to a reduced ability to sustain attention (Gentile, 2011). However, further studies utilizing different methodologies (e.g., behavioral and imagining studies) are needed to investigate the scan-and-shift and arousal hypotheses in relation to attention-related problems. Utilizing cognitive attention and behavioral tasks that are design to assess underlying attentional and executive control and reward processing would allow us to test whether individuals who have higher exposure to fast-paced media actually have difficulty sustaining attention and lower baseline arousal.

Given the high degree of overlap between fast-paced media and violent media, it is not surprising that the statistically unique impact of each was reduced when both were in the model. It remains a bit unclear how best to interpret this. Is it truly the case that violent media are more strongly associated with long-term attention problems than fast-paced media? It is possible, of course, that the evolutionary importance of noticing violence combined with the fact that the most popular entertainment media are both violent and fast-paced explains the present results. Clearly, large-scale longitudinal studies that assess both media pace and media violence exposure are badly needed to help address questions of the uniqueness and direction of these two media variables. Nonetheless, our findings are consistent with Media Effects theory and with prior studies—including longitudinal ones— that have repeatedly found that individuals with higher screen media exposure and higher exposure to violent media content also report higher attention-related problems.

### Limitations

The present findings must be interpreted with several limitations in mind. Our samples of late-adolescent/young adult college students limit the ability to generalize findings to higher-risk and different aged samples. Although the relationship between media effects and attention-related problems in young adults is an important and often overlooked issue, future studies should attempt to replicate these findings with community, clinical, at-risk, and younger samples.

Another limitation is that all measures were based on self-reports. Future research would benefit by using multi-method approaches, including self-reports, peer reports, and behavioral measures of attention processes and impulsivity. Our measurement models included several correlations among residuals, many of which shared method variance. Allowing the residuals to be correlated can improve model fit because the shared variance between indicators is accounted for. It is important to recognize that adding correlated residuals without theoretical rationale can lead to over-fitting and may not generalize to other samples. Lastly, these studies used a cross-sectional correlational design in which all constructs were measured simultaneously, limiting the ability to examine the direction of effects. Long-term experimental and longitudinal-correlational studies are needed to further investigate the causal direction of these relationships. Perhaps media effects are more pronounced in young children, whereas in adolescents and adults we tend to see reciprocal effects of media; in that exposure to fast-paced media in young adults may exacerbate difficulties sustaining attention and exercising inhibitory control, although future studies are needed to clarify these effects.

## Conclusion

Although exposure to fast-paced media appears to have concurrent associations with higher levels of ADHD symptoms, media violence exposure appears to be a stronger predictor of impulsivity. Therefore, it is important to assess media violence when examining media pacing effects in future studies. Such research is important in understanding the impacts of media use.

## Data Availability

Data from the Pilot study cannot be made publicly available, in accordance with our Institutional Review Board regulations, as participants were not informed at the time of the study that their data might be shared on publicly accessible repositories. Data from Studies 1 and 2 are available upon request. The datasets presented in this study can be found in online repositories. Research materials and analysis code are available on OSF. This data can be accessed here: https://osf.io/3pgbt/?view_only=f735dfd3b5904fa68abcde2d56f975e3.

## Footnotes

^1^The proper use and interpretation of background variables has been discussed many times in the media effects domain. For recent discussions see Beyens et al. (2020), Bushman and Anderson (2023), Wright (2021).

^2^Raw data for the Pilot study, conducted over a decade ago, are not available because of changes in IRB policy.

^3^We originally intended to rate the scale on a 7-point scale as done in past studies and in the OSF study materials. However, in study 1 there was a Qualtrics issue which created the 9-point scale (0 = never and 8 = all the time), which is what we report in the manuscript. We corrected the Qualtrics issue in Study 2, resulting in 7-point scales.

^4^Because of this difference in response format for the two violent content items, we tested whether standardizing ratings of the two items prior to averaging them and computing a violence exposure score would change the internal reliability relative to simply averaging the two raw violent content items. The values were similar, so for ease of interpretation, we decided to use the raw average of these two violent items when calculating the violent exposure score for each media type (video games, movies, and TV).

^5^For all three studies, see Supplementary materials (https://osf.io/3pgbt/?view_only=f735dfd3b5904fa68abcde2d56f975e3) for results of all measurement and multiverse analyses not reported in the main article.

^6^For all three studies, see Supplementary materials for detailed description of measurement and structural models.

^7^For all three studies, see Supplementary materials for detailed description structural models and for results of analyses with biological sex added to the model.

^8^Bootstrap estimates yielded similar results to initial models.

^9^However, the fast-paced exposure effects were significant in the “Outliers included” multiverse analyses.
