# Supplementary material for: Fast-paced and violent media exposure are positively associated with ADHD and impulsivity in college students
Source: Front Psychol. 2025 May 12;16:1572895. doi: 10.3389/fpsyg.2025.1572895 (PMC12104283; doi:10.3389/fpsyg.2025.1572895)
Supplement: Supplementary file 1 [file Supplementary_file_1.docx]

Supplemental: Fast-Paced and Violent Media Exposure: Associations with Attention-Related Problems

**CONTENTS**

**Pilot Study 4**

**Methods 4**

***Participants* 4**

***Measures* 4**

*Media Habits Questionnaire description and scoring* 4

*Attention Deficit-Hyperactivity Disorder* 5

*Impulsivity* 5

*Self-Control* 5

***Data Screening and assumptions* 5**

***Planned Analyses* 7**

**Results 8**

*Preliminary Analyses* 8

**Winsorized Measurement Model. 9**

**Winsorized Structural Equation Models. 11**

**Discussion 17**

**Multiverse Structural Equation Models. 19**

*Table S1e: SEM model fit for raw data* 19

*Table S1f: SEM model fit for outliers excluded data* 20

**STUDY 1 21**

**Sample Information. 21**

*Table S2a: Sample demographics and summary statistics for multiverse data sets* 21

*Table S2b: Correlations for the winsorized data set* 21

*Table S2c: Correlations for the multiverse data sets* 22

**Winsorized Measurement Model. 22**

*Initial measurement model description* 22

*Table S2d: Measurement Model Fit* 23

**Winsorized Structural Equation Models. 23**

*Media pacing SEM description* 23

*Combined media pacing and media violence SEM description* 23

*SEM with biological sex description* 24

*SEM with biological sex results* 24

*Alternative combined Media Pacing and Media Violence SEM description and results* 25

*Table S2e: SEM model fit* 26

**Multiverse Structural Equation Models. 27**

*Table S2f: SEM model fit for raw data* 27

*Table S2g: SEM model fit for outliers exclude data* 28

*Figure 2a Media Pacing Only Model* 29

*Figure 2b Combined pacing & violence model with Biological Sex* 30

*Figure 2c Alternative Model* 31

**STUDY 2 32**

**Sample Information. 32**

*Table S3a: Sample demographics and summary statistics for multiverse data sets* 32

*Table S3b: Correlations for the winsorized data set* 33

*Table S3c: Correlations for the multiverse data sets* 34

**Winsorized Measurement Model. 35**

*Initial measurement model description* 35

*Table S3d: Measurement Model Fit* 36

**Winsorized Structural Equation Models. 36**

*Media pacing SEM description* 36

*Combined media pacing and media violence SEM description* 36

*SEM with biological sex description* 36

*SEM with biological sex results* 37

*Alternative combined Media Pacing and Media Violence SEM description and results* 38

*Table S3e: SEM model fit* 39

**Multiverse Structural Equation Models. 40**

*Table S3f: SEM model fit for raw data* 40

*Table S3g: SEM model fit for outliers excluded data* 41

*Figure 3a Media Pacing Only Model* 42

*Figure 3b Combined Sex Pacing and Violence Model with Biological Sex* 43

*Figure 3c Alternative Model* 44

References 45

**PILOT STUDY**

This study assessed three media types (movies, television, and video games), violent media exposure, fast-paced media exposure, and three attention problems (ADHD, self-control, and impulsivity).

**Method**

**Participants**

The initial sample—collected in 2011-2012— included 235 participants (mean age = 19.51 SD = 1.79, 54.7% female, 79.5% Caucasian American, 8.2% Asian American/Pacific Islander, 5.2% Latino American, 3.9% African American, 2.1% more than one ethnicity, 0.4% Native American, and 0.9% reported a race other than one listed.

**Measures**

***Media Habits***

A modified Media Habits Questionnaire (Anderson and Dill, 2000) was used to assess exposure to different media types (video games, television, and movies) and content (violent, fast-paced vs slow-paced). Participants were asked to list their top three frequently played video games, frequently watched television shows, and frequently watched movies and reported how often they played and/or watched each media example. Participants answered one item about violent content (e.g., “How often do players/characters try to physically injure others in this game?”) and one item about video game pacing (e.g., This game is fast-paced”) and responded using a seven-point scale ranging from (1= “Never” to 7 – “All the Time”) for all items. Reliability for the scores was acceptable: media violence α = .75 and fast-paced media α = .70.

***Attention Deficit-Hyperactivity Disorder***

The Adult Attention Deficit-Hyperactivity Disorder Self-Report **(**ASRS, Brevik et al., 2020) assessed symptoms of ADHD. There are 18 items with three subscales: inattentive (e.g., “How often are you distracted by activity or noise around you?”), hyperactive motor (“How often do you fidget or squirm with your hands or feet when you have to sit down for a long time?”), and hyperactive verbal (e.g., “How often do you interrupt others when they are busy?”). Items were rated on a five-point scale (0 = “Never” to 4 = “Very Often”). Items are averaged for a total score. Higher scores represent greater ADHD symptoms. Reliability for the scores was acceptable: inattentive α = .88, hyper-motor α = .68, hyper-verbal α = .76, and total score α = .84.

***Impulsivity***

The Barratt Impulsiveness Scale (Patton et al., 1995) assessed impulsivity. Participants responded to 30 items (e.g., “I do things without thinking.”), rated on a Likert scale (1 = “Rarely/Never” to 4 = “Almost Always/Always”). Items were averaged; higher scores indicate greater impulsivity. Reliability for the scores was acceptable α = .80.

***Self-Control***

The Brief Self-Control Scale **(**Tangney et al., 2004) assessed self-control. Participants responded to 13 items (e.g., “I am good at resisting temptation."). Items were rated on a Likert scale (1= "Not at All" to 5= "Very Much". Several items were reversed scored, then averaged. Higher scores indicate poorer self-control. Reliability was acceptable, α = .83.

**Data Screening and assumptions**

We screened the initial sample (*N* = 235) for careless responders and missing data. Attention checks were not included in this data set, so we identified careless responders by checking for Longstring responses (Desimone et al., 2015). One case was identified as a careless responder and was removed from subsequent analyses.

Data were also evaluated for potential multivariate and univariate outliers and assumptions of regressions. Univariate outliers (values 3 SD from the mean) were identified by inspecting boxplots and z-score deviations from sample means. Multi-variate outliers were assessed using Mahalanobis distance (*p* < .001) and Cook’s value (> 1). Seven univariate outliers and one multivariate outlier were identified.

Analyses were conducted in three ways, consistent with a multiverse approach (Steegan et al., 2016). This multiverse approach resulted in three-overarching analyses: (1) the inclusion of univariate and multivariate outliers, (2) the exclusion of multivariate and univariate outliers, (3) the exclusion of multivariate and transformation of univariate outliers. It was predetermined that if the results did not differ, they would be reported for the model that best fits the data in terms of assumptions. Conversely, variations from the aforementioned analysis would be reported if the results differed.

Regression assumptions were tested for each model: (1) Multicollinearity (Variance Inflation Factor <10 and tolerance scores >.2); (2) Homoscedasticity (standardized residuals were plotted against standardized predicted values and an absence of a funnel or fan shape represented Homoscedasticity); (3) Linearity (standardized residuals were plotted against standardized predicted values and an absence of a curvilinear shape represented linearity); and (4) Normality (P-P plot and histograms were examined for violations). Several media variables were slightly positively skewed, although the values were within recommended guidelines; there were no major violations of assumptions. The data excluding multivariate and but including transformed univariate outliers (e.g., winsorized) best met all the regression assumptions and is therefore reported in subsequent analyses.

**Planned Analyses**

First, we examined measurement models with ADHD symptoms, impulsivity, self-control, media violence, and fast-paced media as observed variables. We examined whether attention-related problems best represented a one or two-factor latent structure. After obtaining an acceptable fit for the measurement model, we examined the structural portion of the model by adding paths from media pacing and media violence to endogenous latent variables. Given the complexity of examining models with items as indicators, we used the total scores for media pacing and media violence in subsequent models.

We conducted several SEMs to examine the associations between media content (e.g., violent and fast-paced media) and attention-related problems. First, we tested the hypothesis that exposure to fast-paced media (e.g., video games, TV, and movies) would be positively associated with attention-related problems. Then we added media violence to the model to test whether pacing and violence were uniquely associated with attention-related problems. We also investigated whether biological sex predicted these effects through a series of exploratory SEMs.

Structural equation models (SEM) were conducted in Mplus Version 8.6 using maximum likelihood robust estimation (Muthen & Muthen, 1998-2011). The χ2, comparative fit index (CFI; recommended CFI value ≥ 0.90), Tucker Lewis index (TLI; recommended value ≥ 0.95), standardized root mean square residual (SRMR; recommended SRMR value ≤ 0.08), and root mean square error of approximation (RMSEA; recommended RMSEA value ≤ 0.06) were used as fit indices to test model fit (Hu & Bentler, 1999; Yuan et al., 2016).

Nested models were compared using the Satorra Bentler Scaled χ2 difference test. A statistically significant χ2 difference test rejects the hypothesis of equal model fit. The model that fits the data best is retained (Kline, 2005). In addition to model fit indices, modification indices were also examined to identify areas of poor fit.

**Results**

**Preliminary Analyses**

In the preliminary analyses, we examined means, standard deviations, and correlations among variables of interest. Table S1a shows the means and standard deviations. Table S1b shows the zero-order correlations among demographic and study variables.

|  | **Winsorized** | | **Raw** | | **Outliers Excluded** | |
| --- | --- | --- | --- | --- | --- | --- |
| **Variable** | **Mean** | ***SD*** | **Mean** | ***SD*** | **Mean** | ***SD*** |
| Media Violence | 11.73 | 5.09 | 11.77 | 5.12 | 11.65 | 5.06 |
| Fast-Paced Media | 13.86 | 4.21 | 13.89 | 4.22 | 13.81 | 4.19 |
| Inattention | 1.84 | 0.55 | 1.85 | 0.57 | 1.82 | 0.52 |
| Hyper-motor | 1.68 | 0.68 | 1.69 | 0.67 | 1.68 | 0.67 |
| Hyper-verbal | 1.46 | 0.73 | 1.46 | 0.74 | 1.45 | 0.71 |
| ADHD Total | 1.66 | 0.50 | 1.72 | 0.50 | 1.70 | 0.46 |
| Self-control | 2.93 | 0.60 | 2.94 | 0.62 | 2.93 | 0.59 |
| Impulsivity | 2.15 | 0.31 | 2.15 | 0.32 | 2.15 | 0.31 |

Table S1a. Descriptive data for multiverse data sets.

Table S1b. Correlations for winsorized data set

|  | 1. | 2. | 3. | 4. | 5. | 6. | 7. | 8. | 9. |
| --- | --- | --- | --- | --- | --- | --- | --- | --- | --- |
| 1.Sex | --- |  |  |  |  |  |  |  |  |
| 2.Media Violence | -.54** | --- |  |  |  |  |  |  |  |
| 3.Fast-Paced Media | -.40** | .83** | --- |  |  |  |  |  |  |
| 4.Inattention | .02 | .13* | .10 | --- |  |  |  |  |  |
| 5.Hyper-Motor | .03 | .09 | .10 | .47** | --- |  |  |  |  |
| 6.Hyper-Verbal | .02 | .07 | .10 | .30** | .36** | --- |  |  |  |
| 7.Adhd Total | .03 | .12 | .13 | .73** | .80** | .76** | --- |  |  |
| 8.Self-Control | -.02 | .21** | .17* | .51** | .36** | .30** | .50** | --- |  |
| 9.Impulsivity | -.02 | .09 | .08 | .54** | .52** | .35** | .61** | .66* | --- |

Notes. Bolded correlations are significant. *** *p < .001, ** p < .01, p < .05,* Sex was coded 0=male, 1=female.

Significant correlations were found among media violence, fast-paced media, inattention, and self-control (ranging from *r* = .13 to .21), partially confirming the main hypotheses. Impulsivity was not significantly correlated with media pacing or media violence. Biological sex was negatively correlated with media violence and fast-paced media, indicating that males, compared to females, reported higher levels of exposure to fast-paced and violent media. The correlations between biological sex and fast-paced media, ADHD symptoms, impulsivity, and self-control were non-significant.

**Winsorized Measurement Model**

Before testing our hypothesis (i.e., the structural portion of the model), we examined the measurement model in a series of confirmatory factor analyses. The initial measurement model included ADHD symptoms, impulsivity, self-control, media violence, and fast-paced media as observed variables. We examined whether ADHD-related problems best represented a one or two-factor latent structure. As can be seen in Table S1c, the results showed that the initial one-factor model (Model 1) would not converge. An initial two-factor model (Model 2) was also a poor fit to the data. We examined modification indices to identify potential areas of ill fit. Additional covariances were added to the models based on modification indices (covariances were added between the following observed variables: hyper-verbal and hyper-motor). Many of these indicators shared method variance (e.g., similarly worded items). The modified one-factor model (Model 3) would not converge. Results showed that the modified two-factor model (Model 4) fit the data well. The Satorra-Bentler Scaled χ2 difference indicated that a modified two-factor structure fit the data as well as the initial two-factor structure. A two-factor model of attention-related problems, with ADHD symptoms as one latent factor and impulsivity (impulsivity and self-control) as the other latent factor, fit the data best.

Table S1c. Measurement Model Fit

| Model | χ2 | DF | *p*-value | RMSEA | CFI | TLI | SRMR | Comparison  Model | χ2 diff (df),  *p* value |
| --- | --- | --- | --- | --- | --- | --- | --- | --- | --- |
| Model 1  1-Factor CFA | Model non-convergence |  |  |  |  |  |  |  |  |
| Model 2  2-Factor CFA | 18.153 | 10 | .059 | .986 | .971 | .783 | .029 |  |  |
| Model 3  1-Factor CFA with mods | Model non-convergence |  |  |  |  |  |  |  |  |
| Model 4  2-Factor CFA with mods | 15.962 | 9 | 0.67 | .058 | .988 | .973 | .027 | Model 2 | 2.181 (1),  *p* = .139 |

**Winsorized Structural Equation Models**

***Does media pacing predict ADHD-related problems?***

For the SEM included two latent factors (ADHD & impulsivity) and two observed variables (media pacing and media violence). There were two path coefficients from media pacing to the two latent variables. Two path coefficients from media violence to the two latent variables were constrained to zero. A residual covariance was added between media pacing and media violence.

Results showed that the model fit (Model 6) the data well, χ2 (11) = 17.352, *p* = .0979, RMSEA = 0.050 [0.000, 0.092], CFI = 0.981, TLI = 0.966, SRMR = 0.031. As shown in Figure S1a, media pacing trended in the hypothesized direction. For ADHD symptoms the association was marginally significant (B = 0.014 [0.000, 0.028], *p* = 0.051, β = 0.149). However, although media pacing was positively correlated with each of the attention problems (significant only for self-control), it did not significantly predict the latent impulsivity factor (B = 0.008 [-0.001, 0.018], *p* = 0.092, β = 0.125).

Figure S1a. Pacing-Only Model 6

Notes: Parameters are standardized slopes. Values in parentheses are SEs.

***Combined Media Pacing and Media Violence Model***

Because we were interested in investigating the simultaneous effects of both media pacing and violence, we examined models with both media variables in the model (Model 7). This SEM included two latent factors (ADHD & impulsivity) and two observed variables (media pacing and media violence). There were four path coefficients from media pacing and media violence to the two latent variables. A residual covariance was added between media pacing and media violence.

Results in Table S1d showed that the model (Model 7) fit the data well, χ2 (9) = 15.962, *p* = 0.0677, RMSEA = 0.058 [0.000, 0.103], CFI = 0.979, TLI = 0.954, SRMR = 0.027. In comparing model fit, the χ2 diff (2) = 1.42, *p* = 0.491, indicated that the pacing-only (Model 6) and combined (Model 7) models had a similar fit.

Table S1d: SEM model fit (N= 233)

| Model | χ2 | DF | p-value | RMSEA | CFI | TLI | SRMR | Comparison  Model | χ2 diff (df), p value |
| --- | --- | --- | --- | --- | --- | --- | --- | --- | --- |
| Model 5 Baseline  2-Factor SEM Fixed | 21.263 | 13 | .068 | .052 | .975 | .962 | .065 | Model 6  Model 7 | 3.89 (2), p =.143  5.33 (4), p = .255 |
| Model 6  2-Factor SEM  Pacing-Only | 17.352 | 11 | .098 | .050 | .981 | .966 | .031 |  |  |
| Model 7  2-Factor SEM Combined | 15.962 | 9 | .068 | .058 | .979 | .954 | .027 | Model 6 | 1.42 (2), p = .491 |
| Model 8  Alt. 2-Factor SEM Combined | 15.962 | 9 | .068 | .058 | .988 | .973 | .027 |  |  |
| Model 9  2-Factor SEM with Sex Fixed | 104.588 | 16 | .001 | .154 | .870 | .773 | .107 |  |  |
| Model 10  2-Factor SEM with Sex-> Violence | 64.249 | 15 | .001 | .119 | .928 | .866 | .088 | Model 9 | 38.614 (1), p = .000 |
| Model 11  2-Factor SEM with Sex-> Violence and Pacing | 22.799 | 14 | .064 | .052 | .987 | .974 | .034 | Model 10 | 40.155 (1), p = .000 |
| Model 12  2-Factor SEM with Sex-> Violence, Pacing, ADHD, Impulsivity | 19.576 | 12 | .076 | .052 | .989 | .974 | .025 | Model 11 | 3.22 (2), p = .199 |

Figure S1b shows that when both predictors were in the model, media pacing was not a significant unique predictor of ADHD symptoms (B = 0.004 [-0.014, 0.034], *p* = 0.767, β = 0.045) or impulsivity (B = 0.000 [-0.018, 0.018], *p* = 0.990, β = 0.000). Media violence also was not a significant unique predictor of either ADHD symptoms (B = 0.010 [-0.014, 0.034], *p* = 0.429, β = 0.125) or impulsivity (B = 0.008 [-0.006, 0.022], *p* = 0.245, β = 0.152.

Figure S1b. Combined Model 7 (*N =* 233)

Notes: Parameters are standardized slopes. Values in parentheses are SEs.

***Combined Media Pacing and Media Violence Models with Biological Sex description and results***

Zero-order correlations (Table S1b) revealed moderate to large effect sizes between biological sex and media pacing and violence and small to moderate effect sizes between media exposure (pacing and violence) and self-control. Therefore, we conducted a series of SEMs investigating the impact of biological sex on ADHD symptoms and impulsivity (e.g., impulsivity and self-control). As shown in Table S1d, in the first model that included sex (Model 9), biological sex was added to a model with the four paths to media violence, media pacing, ADHD symptoms, and impulsivity constrained to zero. In the second model (Model 10), the path from biological sex to media violence was allowed to be freely estimated. In the third model (Model 11), the paths from biological sex to media violence and pacing were freely estimated. In the fourth model (Model 12), the paths from biological sex to media violence, media pacing, ADHD symptoms, and impulsivity were allowed to be freely estimated.

We compared these nested models using the Satorra-Bentler Scaled χ2 difference test. Model fit of the biological sex-constrained model (Model 9) was compared to the model with biological sex and media violence freed (Model 10). The χ2 difference test indicated that the Model 10 was a better fit. This model was compared to Model 11 with paths from biological sex to media violence and media pacing freed. The χ2 difference test indicated that Model 11 was a better fit to the data. Next, we compared Model 11 with a model with paths from biological sex to media violence, media pacing, ADHD symptoms, and impulsivity freely estimated (Model 12). The χ2 difference test indicated that models fit equally well. The more parsimonious model (Model 11), with the path from biological sex to media violence and media pacing, was retained χ2 (14) = 22.799, *p* = 0.064, RMSEA = 0.052 [0.000, 0.089], CFI = 0.987, TLI = 0.974, SRMR = 0.034. Results for Model 11 (Figure S1c) showed that biological sex predicted exposure to fast-paced media (B = -3.403 [-4.416, -2.390], *p* = 0.000, β = -0.403) and media violence (B = -5.485 [-6.619, -4.350], *p* = 0.000), β = -0.537). Results also showed that when both predictors were in the model, media pacing was not a significant unique predictor of ADHD symptoms (B = 0.004 [-0.024, 0.032], *p* = 0.767, β = 0.045) or impulsivity (B = 0.000 [-0.018, 0.018], *p* = 0.999, β = 0.000). Media violence also did not uniquely predict ADHD symptoms (B = 0.010 [-0.014, 0.034], *p* = 0.429, β = 0.125) and impulsivity (B = 0.008 [-0.006, 0.022], *p* = 0.245, β = 0.152).

Figure S1c. Sex Predicting Violence and Pacing Model 11

Notes: Parameters are standardized slopes. Values in parentheses are SEs. Sex was coded 0=male, 1=female.

***Alternative combined Media Pacing and Media Violence SEM description and results***

We conducted an alternative model in which the directionality of the links between the two media exposure variables and the two attention problem latent factors was reversed. This SEM included a two-factor latent (ADHD & impulsivity) and observed variables (media pacing and media violence). There were four path coefficients from the two latent variables (ADHD symptoms and impulsivity) to media pacing and media violence. A residual covariance was added between ADHD symptoms and impulsivity. Given the cross-sectional design, it is not surprising that the model (Model 8, Figure S1d) fit the data well χ2 (9) = 15.962, *p* = 0.0677, RMSEA = 0.058 [0.000, 0.103], CFI = 0.988, TLI = 0.973, SRMR = 0.027. More importantly, in this reversed directionality model, ADHD symptoms did not significantly predict media pacing (B = 1.783 [-4.455, 8.021], *p* = 0.575, β = 0.169) or media violence (B = 1.617 [-5.913, 9.147], *p* = 0.674, β = 0.126). Similarly, impulsivity did not significantly predict media pacing (B =-0.347 [-9.394, 8.701], *p* = 0.940, β = -0.023) or media violence (B = 0.750 [-9.902, 11.403], *p* = 0.890, β = 0.040). Thus, this alternative explanation of the obtained associations was not supported.

Figure S1d. Alternative Combined Model 8

Notes: Parameters are standardized slopes. Values in parentheses are SEs.

**Discussion**

This study found some weak support that exposure to fast-paced media is positively associated with ADHD-like attention problems. When both media pace and violence were in the model, neither was uniquely associated with the attention measures. However, the media variables were highly correlated (*r* = .83). In this pilot study assessment of both pacing and violent content was based on a single item for each of the nine media instances listed by participants. Although the single-item assessing violent content has worked well in many past studies, there are no prior studies of media pacing effects against which to gage the efficacy of a single-item pacing method. In an attempt to improve the distinctiveness of these two conceptual variables, we added additional items. Furthermore, the small effect sizes obtained in the pilot study suggested that a larger sample size may be needed to adequately test these associations.

**Multiverse Structural Equation Models.**

Table S1e: Pilot Study SEM model fit for raw data (N = 234)

| Model | χ2 | DF | *p*-value | RMSEA | CFI | TLI | SRMR | Comparison  Model | χ2 diff (df), *p* value |
| --- | --- | --- | --- | --- | --- | --- | --- | --- | --- |
| Model 1  1-Factor CFA | Model non-convergence |  |  |  |  |  |  |  |  |
| Model 2  2-Factor CFA | 19.022 | 10 | .040 | .062 | .985 | .969 | .029 |  |  |
| Model 3  1-Factor CFA with mods | Model non-convergence |  |  |  |  |  |  |  |  |
| Model 4  2-Factor CFA with mods | 16.555 | 9 | .056 | .060 | .988 | .971 | .027 | Model 2 | 2.440 (1), *p* = .118 |
| Model 5 Baseline  2-Factor SEM Fixed | 23.635 | 13 | .034 | .059 | .969 | .953 | .073 | Model 6  Model 7 | 5.125 (2), *p* =.077  7.088 (4), *p* = .131 |
| Model 6  2-Factor SEM Pacing-Only | 18.441 | 11 | .072 | .054 | .979 | .961 | .031 |  |  |
| Model 7  2-Factor SEM Combined | 16.555 | 9 | .056 | .060 | .978 | .952 | .027 | Model 6 | 1.941 (2), *p* = .378 |
| Model 8  Alt. 2-Factor SEM Combined | 16.555 | 9 | .056 | .060 | .988 | .971 | .027 |  |  |
| Model 9  2-Factor SEM with Sex Fixed | 106.709 | 16 | .000 | .156 | .870 | .772 | .108 |  |  |
| Model 10  2-Factor SEM with Sex-> Violence | 65.856 | 15 | .000 | .120 | .927 | .864 | .089 | Model 9 | 38.532 (1), *p* = .000 |
| Model 11  2-Factor SEM with Sex-> Violence and Pacing | 23.056 | 14 | .059 | .053 | .987 | .974 | .033 | Model 10 | 40.959 (1), *p* = .000 |
| Model 12  2-Factor SEM with Sex-> ADHD, Violence, Pacing, Impulsivity | 20.114 | 12 | .065 | .054 | .988 | .973 | .025 | Model 11 | 2.956 (2), *p* = .228 |

| Model | χ2 | DF | *p*-value | RMSEA | CFI | TLI | SRMR | Comparison  Model | χ2 diff (df), *p* value |
| --- | --- | --- | --- | --- | --- | --- | --- | --- | --- |
| Model 1  1-Factor CFA | Model non-convergence |  |  |  |  |  |  |  |  |
| Model 2  2-Factor CFA | 14.833 | 10 | .138 | .046 | .991 | .982 | .027 |  |  |
| Model 3  1-Factor CFA with mods | Model non-convergence |  |  |  |  |  |  |  |  |
| Model 4  2-Factor CFA with mods | 13.231 | 9 | .152 | .046 | .992 | .982 | .025 | Model 2 | 1.602 (1), *p* = .206 |
| Model 5 Baseline  2-Factor SEM Fixed | 17.056 | 13 | .196 | .037 | .987 | .980 | .054 | Model 6  Model 7 | 2.241 (2), *p* = .326  3.838 (4), *p* = .428 |
| Model 6  2-Factor SEM Pacing-Only | 14.822 | 11 | .191 | .039 | .988 | .977 | .029 |  |  |
| Model 7  2-Factor SEM Combined | 13.231 | 9 | .152 | .046 | .986 | .969 | .025 | Model 5 | 1.591 (2), *p* = .451 |
| Model 8  Alt. 2-Factor SEM Combined | 13.231 | 9 | .152 | .046 | .992 | .982 | .025 |  |  |
| Model 9  2-Factor SEM with Sex Fixed | 101.481 | 16 | .000 | .153 | .868 | .769 | .108 |  |  |
| Model 10  2-Factor SEM with Sex-> Violence | 60.016 | 15 | .000 | .115 | .931 | .870 | .089 | Model 9 | 40.348 (1), *p* = .000 |
| Model 11  2-Factor SEM with Sex-> Violence and Pacing | 19.082 | 14 | .162 | .040 | .992 | .984 | .031 | Model 10 | 39.983 (1), *p* = .000 |
| Model 12  2-Factor SEM with Sex-> Violence, Pacing, ADHD, Impulsivity | 16.092 | 12 | .187 | .039 | .994 | .985 | .023 | Model 11 | 2.984 (2), *p* = .223 |

Table S1f: Pilot Study SEM model fit for outliers exclude data (N = 227)

**Study 1 Sample Information**

|  | **Winsorized** | | **Raw** | | **Outliers Excluded** | |
| --- | --- | --- | --- | --- | --- | --- |
| **Variable** | **Mean** | ***SD*** | **Mean** | ***SD*** | **Mean** | ***SD*** |
| Media Violence | 12.47 | 5.78 | 12.52 | 5.82 | 12.07 | 5.50 |
| Fast-Paced Media | 13.79 | 3.69 | 13.80 | 3.69 | 13.62 | 3.54 |
| Inattention | 1.79 | 0.64 | 1.80 | 0.65 | 1.75 | 0.61 |
| Hyper-motor | 1.83 | 0.77 | 1.84 | 0.79 | 1.80 | 0.76 |
| Hyper-verbal | 1.39 | 0.91 | 1.40 | 0.93 | 1.35 | 0.89 |
| ADHD Total | 1.70 | 0.61 | 1.72 | 0.63 | 1.67 | 0.58 |
| Self-control | 2.88 | 0.64 | 2.90 | 0.65 | 2.85 | 0.62 |
| Impulsivity | 2.14 | 0.37 | 2.14 | 0.37 | 2.11 | 0.35 |
| Negative Urgency | 2.17 | 0.59 | 2.18 | 0.60 | 2.13 | 0.57 |
| Positive Urgency | 1.78 | 0.58 | 1.79 | 0.61 | 1.75 | 0.55 |
| Sensation Seeking | 2.75 | 0.55 | 2.75 | 0.56 | 2.74 | 0.54 |
| Lack of Premeditation | 1.99 | 0.48 | 2.00 | 0.49 | 1.97 | 0.47 |
| Lack of Perseverance | 2.00 | 0.46 | 2.00 | 0.46 | 1.99 | 0.46 |

Table S2a. Descriptive statistics for multiverse data sets.

Table S2b. Correlations for the winsorized data.

|  | 1. | 2. | 3. | 4. | 5. | 6. | 7. | 8. | 9. |
| --- | --- | --- | --- | --- | --- | --- | --- | --- | --- |
| 1.Sex | --- |  |  |  |  |  |  |  |  |
| 2.Media Violence | **-.36**** | --- |  |  |  |  |  |  |  |
| 3.Fast-Paced Media | -.05 | **.56**** | --- |  |  |  |  |  |  |
| 4.Inattention | .01 | **.15**** | .08 | --- |  |  |  |  |  |
| 5.Hyper-motor | **.10*** | **.12*** | **.15**** | **.54*** | --- |  |  |  |  |
| 6.Hyper-verbal | **.11*** | **.10*** | **.15**** | **.50**** | **.50**** | --- |  |  |  |
| 7.ADHD Total | .08 | **.16**** | **.14**** | **.88**** | **.80**** | **.77**** | --- |  |  |
| 8.Self-control | -.01 | **.15**** | .08 | **.66**** | **.38**** | **.41**** | **.62**** | --- |  |
| 9.Impulsivity Total | -.03 | **.19**** | **.15**** | **.54**** | **.34**** | **.44**** | **.54**** | **.71**** | --- |

Notes. Bolded correlations are significant. *** *p < .001, ** p < .01, p < .05*.

Sex was coded 0=male, 1=female

Table S2c. Correlations for the multiverse data sets.

|  | 1. | 2. | 3. | 4. | 5. | 6. | 7. | 8. | 9. |
| --- | --- | --- | --- | --- | --- | --- | --- | --- | --- |
| 1.Sex | --- | **-.35**** | -.05 | .02 | **.11*** | **.10*** | .08 | .00 | -.02 |
| 2.Media Violence | **-.35**** | --- | **.57**** | **.15**** | **.12*** | **.10*** | **.16**** | **.16**** | **.19**** |
| 3.Fast-Paced Media | -.02 | **.53**** | --- | .07 | **.15**** | **.15**** | **.14**** | .08 | **.14**** |
| 4.Inattention | .04 | **.14**** | .10 | --- | **.56**** | **.52**** | **.89**** | **.67**** | **.55**** |
| 5.Hyper-motor | **.11*** | .10 | **.16**** | **.52**** | --- | **.52**** | **.81**** | **.39**** | **.35**** |
| 6.Hyper-verbal | **.10*** | .08 | **.13**** | **.48**** | **.48**** | --- | **.78**** | **.42**** | **.45**** |
| 7.ADHD Total | .08 | **.14**** | **.15**** | **.87**** | **.79**** | **.76**** | --- | **.62**** | **.56**** |
| 8.Self-control | -.01 | **.14**** | .09 | **.64**** | **.34**** | **.39**** | **.62**** | ---- | **.72**** |
| 9.Impulsivity | -.03 | **.16**** | .**12*** | **.53**** | **.31**** | **.42**** | **.54**** | **.70**** | --- |

Notes. Bolded correlations are significant. *** *p < .001, ** p < .01, p < .05.* Sex was coded 0=male, 1=female. Raw data (N = 442) is above the diagonal and data without outliers (*N* = 412) are below the diagonal.

**Winsorized Measurement Model.**

***Initial measurement model description***

The initial measurement model included ADHD symptoms, impulsivity, self-control, media violence, and fast-paced media as observed variables. We examined whether ADHD-related problems best represented a one or two-factor latent structure. The results showed that the initial one-factor model (Model 1) was a poor fit to the data. An initial two-factor model (Model 2) was also a poor fit to the data. The Satorra-Bentler Scaled χ2 difference indicated that a two-factor structure fits the data best compared to a one-factor structure.

We examined modification indices to identify potential areas of ill fit. Additional covariances were added to the models based on modification indices (covariances were added between the following observed variables: negative and positive urgency, hyper-verbal and hyper-motor, lack of premeditation and lack of perseverance, and sensation seeking and lack of perseverance). Many of these indicators shared method variance (e.g., similarly worded items). The modified one-factor model (Model 3) provided an adequate fit. Results showed that the modified two-factor model (Model 4) fit the data well. The Satorra-Bentler Scaled χ2 difference indicated that a modified two-factor structure fit the data best compared to a one-factor structure.

Table S2d. Measurement Model Fit

| Model | χ2 | DF | *p*-value | RMSEA | CFI | TLI | SRMR | Comparison  Model | χ2 diff (df),  *p* value |
| --- | --- | --- | --- | --- | --- | --- | --- | --- | --- |
| Model 1  1-Factor CFA | 432.440 | 43 | .001 | .142 | .766 | .701 | .075 |  |  |
| Model 2  2-Factor CFA | 355.084 | 40 | .001 | .134 | .806 | .733 | .068 | Model 1 | 85.729 (3),  *p* = .001 |
| Model 3  1-Factor CFA with mods | 187.510 | 39 | .001 | 0.93 | .909 | .871 | .055 |  |  |
| Model 4  2-Factor CFA with mods | 131.463 | 36 | .001 | .078 | .941 | .910 | .052 | Model 3 | 56.097 (3),  *p* = .001 |

**Winsorized Structural Equation Models.**

***Media pacing SEM description***

The SEM included two latent factors (ADHD & impulsivity) and two observed variables (media pacing and media violence). There were two path coefficients from media pacing to the two latent variables. Two path coefficients from media violence to the two latent variables were constrained to zero. A residual covariance was added between media pacing and media violence.

***Combined media pacing and media violence SEM description***

This SEM included two latent factors (ADHD & impulsivity) and two observed variables (media pacing and media violence). There were four path coefficients from media pacing and media violence to the two latent variables. A residual covariance was added between media pacing and media violence.

***SEM with biological sex description***

Zero-order correlations revealed a moderate effect size between biological sex and media violence and some small effect sizes with aspects of ADHD symptoms and impulsivity. Therefore, we conducted a series of SEMs investigating the impact of biological sex on ADHD symptoms and impulsivity. In the first model (Model 9), biological sex was added to the model, with the four paths to media violence, media pacing, ADHD symptoms, and impulsivity constrained to zero. In the second model (Model 10), the path from biological sex to media violence was allowed to be freely estimated. In the third model (Model 11), the paths from biological sex to media violence and pacing were freely estimated. In the fourth model (Model 12), the paths from biological sex to media violence, media pacing, ADHD symptoms, and impulsivity were allowed to be freely estimated.

***SEM with biological sex results***

We compared these nested models using the Satorra-Bentler Scaled χ2 difference test. We compared the model fit of the biological sex-constrained model (Model 9) and the model with biological sex and media violence freed (Model 10). The χ2 difference test indicated that Model 10 was a better fit to the data. This model (Model 10) was then compared to the model with paths from biological sex to media violence and media pacing freed (Model 11). The χ2 difference test indicated that models fit equally well. The more parsimonious model (Model 10) with the path from biological sex to media violence was retained. Next, we compared Model 10 with a model with paths from biological sex to media violence, media pacing, ADHD symptoms, and impulsivity freely estimated (Model 12). The χ2 difference test indicated that the models fit equally well. The more parsimonious model (Model 10) with the path from biological sex to media violence was retained. The model adequately fit the data, χ2 (46) = 188.999, p < 0.001, RMSEA = 0.084 [0.072, 0.097], CFI = 0.919, TLI = 0.884, SRMR = 0.062. Results for Model 10 (Figure S6) showed that biological sex predicted exposure to media violence (B = -3.959 [-4.813, -3.104], *p* = 0.000), β = -0.334). Results also showed that when both media predictors are in the model, media pacing was not a significant unique predictor of ADHD symptoms (B = 0.003 [-0.017, 0.023], *p* = 0.776, β = 0.018) or of impulsivity (B = 0.000 [-0.015, 0.015], *p* = 0.998, β = 0.000). Media violence was a significant predictor of ADHD symptoms (B = 0.015 [0.003, 0.028], *p* = 0.017, β = 0.148) and of impulsivity (B = 0.012 [0.003, 0.021], *p* = 0.012, β = 0.156).

***Alternative combined Media Pacing and Media Violence SEM description and results***

This SEM included a two-factor latent (ADHD & impulsivity) and observed variables (media pacing and media violence). There were four path coefficients from the two latent variables (ADHD symptoms and impulsivity) to media pacing and media violence. A residual covariance was added between ADHD symptoms and impulsivity. The model (Model 8, Figure S2b) fit the data well, χ2 (36) =131.463, *p* < 0.001, RMSEA = 0.078 [0.064, 0.092], CFI = 0.941 TLI = 0.910, SRMR = 0.052. More importantly, in this reversed directionality model, ADHD symptoms was not a significant predictor of media pacing (B = 0.518 [-0.791, 1.827], *p* = 0.438, β = 0.083) or of media violence (B = 0.868 [-0.963, 2.700], *p* = 0.353), β = 0.089). Similarly, impulsivity was not a significant predictor of media pacing (B = 0.208 [-1.512, 1.928], *p* = 0.813, β = 0.025) or of media violence (B = 1.236 [-1.157, 3.629], *p* = 0.311, β = 0.094). Thus, this alternative explanation of the obtained associations was not supported.

Table S2e. Model Fit for Study 1

| Model | χ2 | DF | *p-value* | RMSEA | CFI | TLI | SRMR | Comparison  Model | χ2 diff (df), p value |
| --- | --- | --- | --- | --- | --- | --- | --- | --- | --- |
| Model 5 Baseline  2-Factor SEM Fixed | 143.085 | 40 | *.001* | .077 | .931 | .907 | .072 | Model 6  Model 7 | 3.768 (2), p = .152  11.728 (4), p = .019 |
| Model 6  2-Factor SEM  Pacing-Only | 139.292 | 38 | *.001* | .078 | .932 | .903 | .059 |  |  |
| Model 7  2-Factor SEM Combined | 131.463 | 36 | *.001* | .078 | .936 | .904 | .052 | Model 6 | 7.729 (2), p = .021 |
| Model 8  Alt. 2-Factor SEM Combined | 131.463 | 36 | *.001* | .078 | .941 | .910 | .052 |  |  |
| Model 9  2-Factor SEM with Sex Fixed | 255.750 | 47 | *.001* | .101 | .882 | .835 | .070 |  |  |
| Model 10  2-Factor SEM with Sex-> Violence | 188.999 | 46 | *.001* | .084 | .919 | .884 | .062 | Model 9 | 59.405 (1), p = .000 |
| Model 11  2-Factor SEM with Sex-> Violence and Pacing | 187.603 | 45 | *.001* | .085 | .920 | .882 | .062 | Model 10 | 1.640 (1), p = .200 |
| Model 12  2-Factor SEM with Sex-> Violence, Pacing, ADHD, Impulsivity | 184.942 | 43 | *.001* | .087 | .920 | .877 | .057 | Model 10 | 3.826 (3), p = .281 |

**Multiverse Structural Equation Models**

Table S2f. Model fit for Study 1 (Raw data *N* = 442)

| Model | χ2 | DF | *p*-value | RMSEA | CFI | TLI | SRMR | Comparison  Model | χ2 diff (df),  *p-*value |
| --- | --- | --- | --- | --- | --- | --- | --- | --- | --- |
| Model 1  1-Factor CFA | 424.413 | 43 | .000 | .142 | .775 | .712 | .074 |  |  |
| Model 2  2-Factor CFA | 344.931 | 40 | .000 | .131 | .820 | .753 | .066 | Model 2 | 95.573 (3), *p* = .000 |
| Model 3  1-Factor CFA with mods | 191.859 | 39 | .000 | .094 | .910 | .873 | .054 |  |  |
| Model 4  2-Factor CFA with mods | 128.795 | 36 | .000 | .076 | .945 | .916 | .050 | Model 3 | 61.931 (3), *p* = .000 |
| Model 5 Baseline  2-Factor SEM Fixed | 141.599 | 40 | .000 | .076 | .935 | .912 | .071 | Model 6  Model 7 | 3.788 (2), *p* = .150  12.900 (4), *p* = .012 |
| Model 6  2-Factor SEM  Pacing-Only | 137.871 | 38 | .000 | .077 | .936 | .909 | .058 |  |  |
| Model 7  2-Factor SEM Combined | 128.795 | 36 | .000 | .076 | .940 | .911 | .050 | Model 6 | 8.869 (2), *p* = .012 |
| Model 8  Alt. 2-Factor SEM Combined | 128.795 | 36 | .000 | .076 | .945 | .916 | .050 |  |  |
| Model 9  2-Factor SEM with Sex Fixed | 245.95 | 47 | .000 | .098 | .889 | .846 | .068 |  |  |
| Model 10  2-Factor SEM with Sex-> Violence | 183.141 | 46 | .000 | .082 | .925 | .893 | .061 | Model 9 | 55.775 (1), *p* = .000 |
| Model 11  2-Factor SEM with Sex-> Violence and Pacing | 181.778 | 45 | .000 | .083 | .925 | .891 | .061 | Model 10 | 1.624 (1), *p* = .202 |
| Model 12  2-Factor SEM with Sex-> Violence, Pacing, ADHD, Impulsivity | 178.515 | 43 | .000 | .084 | .926 | .887 | .055 | Model 10 | 4.520 (3), *p* = .210 |

Table S2g. Model fit for Study 1 (Outliers Excluded *N* = 412)

| Model | χ2 | DF | *p*-value | RMSEA | CFI | TLI | SRMR | Comparison  Model | χ2 diff (df), *p-*value |
| --- | --- | --- | --- | --- | --- | --- | --- | --- | --- |
| Model 1  1-Factor CFA | 375.292 | 43 | .000 | .137 | .764 | .698 | .074 |  |  |
| Model 2  2-Factor CFA | 314.701 | 40 | .000 | .129 | .805 | .732 | .066 | Model 1 | 73.106 (3), *p* = .000 |
| Model 3  1-Factor CFA with mods | 172.193 | 39 | .000 | .091 | .905 | .876 | .054 |  |  |
| Model 4  2-Factor CFA with mods | 121.114 | 36 | .000 | .076 | .940 | .9908 | .050 | Model 3 | 50.808 (3), *p* = .000 |
| Model 5 Baseline  2-Factor SEM Fixed | 130.992 | 40 | .000 | .074 | .930 | .906 | .068 | Model 6  Model 7 | 4.887 (2), *p* = .087  9.955 (4), *p* = .041 |
| Model 6  2-Factor SEM  Pacing-Only | 126.123 | 38 | .000 | .075 | .932 | .904 | .055 |  |  |
| Model 7  2-Factor SEM Combined | 121.114 | 36 | .000 | .076 | .935 | .902 | .050 | Model 6 | 5.066 (2), *p* = .079 |
| Model 8  Alt. 2-Factor SEM Combined | 121.114 | 36 | .000 | .076 | .940 | .908 | .050 |  |  |
| Model 9  2-Factor SEM with Sex Fixed | 232.220 | 47 | .000 | .098 | .880 | .831 | .067 |  |  |
| Model 10  2-Factor SEM with Sex-> Violence | 169.475 | 46 | .000 | .081 | .920 | .885 | .058 | Model 9 | 53.623(1), *p* = .000 |
| Model 11  2-Factor SEM with Sex-> Violence and Pacing | 168.969 | 45 | .000 | .082 | .919 | .882 | .058 | Model 10 | 0.878 (1), *p* = .348 |
| Model 12  2-Factor SEM with Sex-> Violence, Pacing, ADHD, Impulsivity | 167.097 | 43 | .000 | .084 | .919 | .876 | .055 | Model 10 | 2.439 (3), *p* = .486 |

These are Figures for Study 1 (winsorized data)

Figure S2a. Pacing-Only Model 6

Notes: Parameters are standardized slopes. Values in parentheses are SEs. Multiverse approach: Media Pace->ADHD was significant in Outliers included and Excluded data sets. Media Pace->Impulsivity was significant in Outliers included data set.

Figure S2b. Sex Predicting Violence Model 10

Notes: Parameters are standardized slopes. Values in parentheses are SEs. Sex was coded 0=male, 1=female. Multiverse approach: Media Pace->ADHD was significant in Outliers included data set. Media Violence->ADHD and Impulsivity was not significant in Outliers included and Excluded data sets.

Figure S2c. Alternative Combined Model 8

Notes: Parameters are standardized slopes. Values in parentheses are SEs. Multiverse approach: Impulsivity->Media Violence was significant in Outliers included data set.

**Study 2 Sample Information.**

|  | **Winsorized** | | **Raw** | | **Outliers Excluded** | |
| --- | --- | --- | --- | --- | --- | --- |
| **Variable** | **Mean** | ***SD*** | **Mean** | ***SD*** | **Mean** | ***SD*** |
| Media Violence  Z-score | -0.04 | 0.59 | -0.02 | .060 | -0.07 | 0.57 |
| Fast-Paced Media  Z-score | 0.01 | 0.58 | 0.17 | 0.59 | -0.01 | 0.58 |
| Inattention | 2.05 | 0.67 | 2.07 | 0.68 | 2.05 | 0.66 |
| Hyper-motor | 2.08 | 0.77 | 2.11 | 0.78 | 2.09 | 0.76 |
| Hyper-verbal | 1.56 | 0.90 | 1.58 | 0.91 | 1.55 | 0.88 |
| ADHD Total | 1.95 | 0.62 | 1.97 | 0.64 | 1.95 | 0.61 |
| Self-control | 2.92 | 0.60 | 2.94 | 0.62 | 2.91 | 0.59 |
| Impulsivity | 2.05 | 0.35 | 2.06 | 0.37 | 2.04 | 0.35 |
| Negative Urgency | 2.16 | 0.69 | 2.19 | 0.71 | 2.15 | 0.69 |
| Positive Urgency | 1.83 | 0.60 | 1.86 | 0.63 | 1.81 | 0.58 |
| Sensation Seeking | 2.57 | 0.67 | 2.58 | 0.67 | 2.57 | 0.67 |
| Lack of Premeditation | 1.84 | 0.51 | 1.85 | 0.53 | 1.82 | 0.48 |
| Lack of Perseverance | 1.83 | 0.48 | 1.83 | 0.50 | 1.83 | 0.48 |

Table S3a. Descriptive statistics for multiverse data sets.

Table S3b. Correlations for the winsorized data.

|  | 1. | 2. | 3. | 4. | 5. | 6. | 7. | 8. | 9. |
| --- | --- | --- | --- | --- | --- | --- | --- | --- | --- |
| 1.Sex | --- |  |  |  |  |  |  |  |  |
| 2.Media Violence | **-.26**** | --- |  |  |  |  |  |  |  |
| 3.Fast-Paced Media | **.11*** | **.58**** | --- |  |  |  |  |  |  |
| 4.Inattention | **.10*** | **.11*** | **.15**** | --- |  |  |  |  |  |
| 5.Hyper-motor | **.15**** | **.11*** | **.17**** | **.57**** | --- |  |  |  |  |
| 6.Hyper-verbal | **.18**** | .05 | **.16**** | **.49**** | **.49**** | --- |  |  |  |
| 7.ADHD Total | **.16**** | **.11*** | **.19**** | **.89**** | **.81**** | **.75**** | --- |  |  |
| 8.Self-control | -.03 | **.23**** | **.19**** | **.55**** | **.32**** | **.34*** | **.51**** | -- |  |
| 9.Impulsivity Total | **-.11*** | **.11*** | .03 | **.38**** | **.21**** | **.30**** | **.37**** | **.58**** | --- |

Notes. Bolded correlations are significant. *** *p < .001, ** p < .01, p < .05*. Sex was coded 0=male, 1=female.

Table S3c. Correlations for multiverse data sets.

|  | 1. | 2. | 3. | 4. | 5. | 6. | 7. | 8. | 9. |
| --- | --- | --- | --- | --- | --- | --- | --- | --- | --- |
| 1.Sex | --- | **-.26**** | **.11*** | **.10*** | **.16*** | **.18**** | **.16**** | -.02 | -.09 |
| 2.Media Violence | **-.25**** | --- | **.58**** | **.10*** | **.10*** | .04 | **.10*** | **.25**** | **.11*** |
| 3.Fast-Paced Media | **.14**** | **.57**** | --- | **.15**** | **.17**** | **.15**** | **.19**** | **.20**** | .07 |
| 4.InAttention | .09 | **.15**** | **.16**** | --- | **.59**** | **.51**** | **.90**** | **.54**** | **.41**** |
| 5.Hyper Motor | **.14**** | **.13**** | **.18**** | .56** | --- | **.51**** | **.82**** | **.31**** | **.24**** |
| 6.Hyper Verbal | **.17**** | .05 | **.16**** | **.49**** | **.48**** | --- | **.77**** | **.32**** | **.32**** |
| 7.ADHD-Total | **.15**** | **.14**** | **.20**** | **.89**** | **.80**** | **.75**** | --- | **.49**** | **.40**** |
| 8.Self Control | -.02 | **26**** | **.19*** | **.54**** | **.31**** | **.34**** | **.51**** | ---- | **.57**** |
| 9.Impulsivity | **-.10*** | **.12**** | .03 | **.36**** | **.19**** | **.28**** | **.35**** | **.57**** | --- |

Notes. Bolded correlations are significant. *** *p < .001, ** p < .01, p < .05.* Sex was coded 0=male, 1=female.

Raw data (N = 468) are above the diagonal and data without outliers (*N* = 435) are below the diagonal.

**Winsorized Measurement Model.**

***Initial measurement model description***

The initial measurement model included ADHD symptoms, impulsivity, self-control, media violence, and fast-paced media as observed variables. We examined whether ADHD-related problems best represented a one or two-factor latent structure. The results showed that the initial one-factor model (Model 1) was a poor fit to the data. An initial two-factor model (Model 2) was also a poor fit to the data. The Satorra-Bentler Scaled χ2 difference test indicated that a two-factor structure fits the data best compared to a one-factor structure.

We examined modification indices to identify potential areas of ill fit. Additional covariances were added to the models based on modification indices (covariances were added between the following observed variables: negative and positive urgency, hyper-verbal and hyper-motor, lack of premeditation and lack of perseverance, and sensation seeking and lack of perseverance). Many of these indicators shared method variance (e.g., similarly worded items). The modified one-factor model (Model 3) provided adequate fit. Results showed that the modified two-factor model (Model 4) fit the data well. The Satorra-Bentler Scaled χ2 difference test indicated that a modified two-factor structure fit the data best compared to a one-factor structure.

Table S3d. Measurement Model Fit

| Model | χ2 | DF | *p*-value | RMSEA | CFI | TLI | SRMR | Comparison  Model | χ2 diff (df),  *p* value |
| --- | --- | --- | --- | --- | --- | --- | --- | --- | --- |
| Model 1  1-Factor CFA | 431.394 | 43 | .001 | .141 | .686 | .598 | .088 |  |  |
| Model 2  2-Factor CFA | 308.647 | 40 | .001 | .121 | .783 | .701 | .077 | Model 1 | 157.712 (3),  *p* = .001 |
| Model 3  1-Factor CFA with mods | 248.153 | 39 | .001 | .108 | .831 | .761 | .064 |  |  |
| Model 4  2-Factor CFA with mods | 153.706 | 36 | .001 | .085 | .905 | .854 | .058 | Model 3 | 81.499 (3),  *p* = .001 |

**Winsorized Structural Equation Models.**

***Media pacing SEM description***

The SEM included two latent factors (ADHD & impulsivity) and two observed variables (media pacing and media violence). There were two path coefficients from media pacing to the two latent variables. Two path coefficients from media violence to the two latent variables were constrained to zero. A residual covariance was added between media pacing and media violence.

***Combined media pacing and media violence SEM description***

This SEM included two latent factors (ADHD & impulsivity) and two observed variables (media pacing and media violence). There were four path coefficients from media pacing and media violence to the two latent variables. A residual covariance was added between media pacing and media violence.

***SEM with biological sex description***

Zero-order correlations revealed a moderate effect size between biological sex, media pacing, and media violence and some small effect sizes with aspects of ADHD symptoms and impulsivity. Therefore, we conducted a series of SEMs investigating the impact of biological sex on ADHD symptoms and impulsivity. In the first model (Model 9), biological sex was added to the model, with the four paths to media violence, media pacing, ADHD symptoms, and impulsivity constrained to zero. In the second model (Model 10), the path from biological sex to media violence was allowed to be freely estimated. In the third model (Model 11), the paths from biological sex to media violence and pacing were freely estimated. In the fourth model (Model 12), the paths from biological sex to media violence, media pacing, ADHD symptoms, and impulsivity were allowed to be freely estimated.

***SEM with biological sex results***

We compared these nested models using the Satorra-Bentler Scaled χ2 difference test. We compared the model fit of the biological sex-constrained model (Model 9) and the model with biological sex and media violence freed (Model 10). The χ2 difference test indicated that the model with the path from biological sex to media violence was a better fit to the data. This model (Model 10) was then compared to the model with paths from biological sex to media violence and media pacing freed (Model 11). The χ2 difference test indicated that Model 11 was a better fit to the data. Next, we compared Model 11 with a model with paths from biological sex to media violence, media pacing, ADHD symptoms, and impulsivity freely estimated (Model 12). The χ2 difference test indicated that Model 12 fits the data best. Model 12, with paths from biological sex to media violence, media pacing, ADHD symptoms, and impulsivity freely estimated, was retained. The model adequately fit the data, χ2 (43) = 192.399, p < 0.001, RMSEA = 0.088 [0.075, 0.101], CFI = 0.889, TLI = 0.830, SRMR = 0.062. Results for Model 12 (Figure S9) showed that biological sex predicted exposure to fast-paced media (B = 0.133 [0.023, 0.243], *p* = 0.017, β = 0.110), media violence (B = -0.325 [-0.474, -0.211], *p* = 0.000, β = -0.262), ADHD symptoms (B = 0.190 [0.047, 0.333], *p* = 0.009), β = 0.152), but not impulsivity (B = 0.004 [-0.089, 0.098], *p* = 0.925, β = 0.006). Results also showed that when biological sex is added to the model, media pacing no longer predicted ADHD symptoms (B = 0.113 [-0.026, 0.252], *p* = 0.111, β = 0.110) or impulsivity (B = 0.047 [-0.041, 0.135], *p* = 0.293, β = 0.071). Media violence was a significant predictor of impulsivity (B = 0.129 [0.037, 0.221], *p* = 0.006, β = 0.199) but not ADHD symptoms (B = 0.105 [-0.036, 0.246], *p* = 0.145, β = 0.104).

***Alternative combined Media Pacing and Media Violence SEM description and results***

This SEM included a two-factor latent (ADHD & impulsivity) and observed variables (media pacing and media violence). There were four path coefficients from the two latent variables (ADHD symptoms and impulsivity) to media pacing and media violence. A residual covariance was added between ADHD symptoms and impulsivity. The model (Model 8, Figure S3c) was of adequate fit, χ2 (36) = 153.706, *p* < 0.001, RMSEA = 0.085 [0.071, 0.099], CFI = 0.905 TLI = 0.854, SRMR = 0.058. More importantly, in this reversed directionality model, ADHD symptoms was not a significant predictor of media pacing (B = 0.092 [-0.059, 0.242], *p* = 0.233, β = 0.097) or media violence (B = -0.059 [-0.214, 0.096], *p* = 0.454, β = -0.062). Similarly, impulsivity was not a significant predictor of media pacing (B = 0.189 [-0.060, 0.438], *p* = 0.136, β = 0.123). However, impulsivity was a predictor of media violence (B = 0.443 [0.180, 0.705], *p* = 0.001, β = 0.282). Thus, this alternative explanation of the obtained associations was partially disconfirmed.

Table S3e. SEM Model Fit

| Model | χ2 | DF | p-value | RMSEA | CFI | TLI | SRMR | Comparison  Model | χ2 diff (df), p value |
| --- | --- | --- | --- | --- | --- | --- | --- | --- | --- |
| Model 5 Baseline  2-Factor SEM Fixed | 179.075 | 40 | .001 | .087 | .870 | .825 | .075 | Model 6  Model 7 | 14.705 (2), p =.001  25.731 (4), p = .001 |
| Model 6  2-Factor SEM  Pacing-Only | 164.718 | 38 | .001 | .086 | .882 | .832 | .059 |  |  |
| Model 7  2-Factor SEM Combined | 153.706 | 36 | .001 | .085 | .890 | .835 | .058 | Model 6 | 11.085 (2), p = .004 |
| Model 8  Alt. 2-Factor SEM Combined | 153.706 | 36 | .001 | .085 | .905 | .854 | .058 |  |  |
| Model 9  2-Factor SEM with Sex Fixed | 278.544 | 47 | .001 | .105 | .828 | .758 | .074 |  |  |
| Model 10  2-Factor SEM with Sex-> Violence | 206.018 | 46 | .001 | .088 | .881 | .829 | .069 | Model 9 | 79.368 (1), p = .000 |
| Model 11  2-Factor SEM with Sex-> Violence and Pacing | 200.554 | 45 | .001 | .088 | .884 | .830 | .067 | Model 10 | 5.356 (1), p = .021 |

**Multiverse Structural Equation Models.**

Table S3f. SEM Model Fit (Raw data *N* = 468)

| Model | χ2 | DF | *p*-value | RMSEA | CFI | TLI | SRMR | Comparison  Model | χ2 diff (df), *p* value |
| --- | --- | --- | --- | --- | --- | --- | --- | --- | --- |
| Model 1  1-Factor CFA | 431.282 | 43 | .000 | .139 | .692 | .606 | .085 |  |  |
| Model 2  2-Factor CFA | 303.484 | 40 | .000 | .119 | .791 | .713 | .073 | Model 2 | 122.837 (3), *p* = .000 |
| Model 3  1-Factor CFA with mods | 246.470 | 39 | .000 | .107 | .836 | .768 | .062 |  |  |
| Model 4  2-Factor CFA with mods | 156.576 | 36 | .000 | .084 | .906 | .856 | .055 | Model 3 | 74.334 (3), *p* = .000 |
| Model 5 Baseline  2-Factor SEM Fixed | 182.429 | 40 | .000 | .087 | .871 | .826 | .074 | Model 6  Model 7 | 16.222 (2), *p* = .000  27.392 (4), *p* = .000 |
| Model 6  2-Factor SEM Pacing-Only | 166.219 | 38 | .000 | .085 | .884 | .835 | .055 |  |  |
| Model 7  2-Factor SEM Combined | 154.576 | 36 | .000 | .084 | .893 | .839 | .055 | Model 6 | 11.385 (2), *p* = .003 |
| Model 8  Alt. 2-Factor SEM Combined | 154.576 | 36 | .000 | .084 | .906 | .856 | .055 |  |  |
| Model 9  2-Factor SEM with Sex Fixed | 281.619 | 47 | .000 | .104 | .830 | .762 | .072 |  |  |
| Model 10  2-Factor SEM with Sex-> Violence | 210.364 | 46 | .000 | .088 | .881 | .830 | .067 | Model 9 | 74.748 (1), *p* = .000 |
| Model 11  2-Factor SEM with Sex-> Violence and Pacing | 204.618 | 45 | .000 | .088 | .885 | .831 | .064 | Model 10 | 5.556 (1), *p* = .018 |
| Model 12  2-Factor SEM with Sex-> ADHD, Violence, Pacing, Impulsivity | 196.285 | 43 | .000 | .088 | .889 | .830 | .059 | Model 11 | 8.287 (2), *p* = .016 |

Table S3g. SEM Model fit (Outliers Excluded *N* = 435)

| Model | χ2 | DF | *p*-value | RMSEA | CFI | TLI | SRMR | Comparison  Model | χ2 diff (df), *p* value |
| --- | --- | --- | --- | --- | --- | --- | --- | --- | --- |
| Model 1  1-Factor CFA | 405.307 | 43 | .000 | .139 | .681 | .593 | .088 |  |  |
| Model 2  2-Factor CFA | 281.295 | 40 | .000 | .118 | .788 | .708 | .075 | Model 2 | 132.458 (3), *p* = .000 |
| Model 3  1-Factor CFA with mods | 233.042 | 39 | .000 | .107 | .829 | .759 | .065 |  |  |
| Model 4  2-Factor CFA with mods | 143.473 | 36 | .000 | .083 | .906 | .856 | .056 | Model 3 | 80.115 (3), *p* = .000 |
| Model 5 Baseline  2-Factor SEM Fixed | 172.024 | 40 | .000 | .087 | .867 | .821 | .076 | Model 6  Model 7 | 15.534 (2), *p* = .000  28.769 (4), *p* = .000 |
| Model 6  2-Factor SEM  Pacing-Only | 156.986 | 38 | .000 | .085 | .880 | .830 | .058 |  |  |
| Model 7  2-Factor SEM Combined | 143.473 | 36 | .000 | .083 | .892 | .838 | .056 | Model 6 | 14.265 (2), *p* = .001 |
| Model 8  Alt. 2-Factor SEM Combined | 143.473 | 36 | .000 | .083 | .906 | .856 | .056 |  |  |
| Model 9  2-Factor SEM with Sex Fixed | 259.203 | 47 | .000 | .102 | .829 | .760 | .071 |  |  |
| Model 10  2-Factor SEM with Sex-> Violence | 192.682 | 46 | .000 | .086 | .882 | .831 | .068 | Model 9 | 77.321(1), *p* = .000 |
| Model 11  2-Factor SEM with Sex-> Violence and Pacing | 183.976 | 45 | .000 | .085 | .888 | .836 | .064 | Model 10 | 8.101 (1), *p* = .004 |
| Model 12  2-Factor SEM with Sex-> Violence, Pacing, ADHD, Impulsivity | 176.241 | 43 | .000 | .085 | .893 | .835 | .060 | Model 11 | 7.675 (2), *p* = .022 |

These are Figures for Study 2 (winsorized data)

Figure S3a. Pacing-Only Model 6

Notes: Parameters are standardized slopes. Values in parentheses are SEs.

Figure S3b. Sex Predicting Violence, Pacing, ADHD, and Impulsivity Model 12

Notes: Parameters are standardized slopes. Values in parentheses are SEs. Sex was coded 0=male, 1=female. Multiverse approach: Media Violence->ADHD was significant in Outliers excluded data set.

Figure S3c. Alternative Combined Model 8

Notes: Parameters are standardized slopes. Values in parentheses are SEs.

**References**

Desimone, J.A., Hamrs, P.D., & Desimone, A.J. (2015). Best practice recommendations for data

screening. *Journal of Organizational Behavior.* 36, 171-181

Patton, J. H., Stanford, M. S., & Barratt, E. S. (1995). Factor structure of the Barratt impulsiveness scale. Journal of clinical psychology, 51(6), 768-774.
